# A temperature-driven DNA discrimination strategy to distinguish *E. coli* DNA and phage 5hmC-modified DNA

**DOI:** 10.1093/nar/gkaf501

**Published:** 2025-06-11

**Authors:** Yue Kang, Yahui Liu, Haolong Zhou, Biyun Ma, Huan Chen, Kaining Zhang, Yawen Wang, Chengpeng Fan, Huaiyu Yang, Yingqi Xu, Steve Matthews, Shuai Yuan, Yan Li, Bing Liu

**Affiliations:** Department of Infectious Diseases, the First Affiliated Hospital of Xi’an Jiaotong University, Shaanxi 710061, China; Key Laboratory of Surgical Critical Care and Life Support, Biobank, Centre for Biobank and Advanced Medical Research of Shaanxi Province, the First Affiliated Hospital of Xi’an Jiaotong University, Shaanxi 710061, China; Department of Pathogen Biology, School of Basic Medicine, Tongji Medical College and State Key Laboratory for Diagnosis and Treatment of Severe Zoonotic Infectious Diseases, Huazhong University of Science and Technology, 13 Hangkong Road, Wuhan 430030, China; State Key Laboratory of Virology and Biosafety, Wuhan Institute of Virology, Chinese Academy of Sciences, Wuhan, Hubei 430071, China; Department of Infectious Diseases, the First Affiliated Hospital of Xi’an Jiaotong University, Shaanxi 710061, China; Key Laboratory of Surgical Critical Care and Life Support, Biobank, Centre for Biobank and Advanced Medical Research of Shaanxi Province, the First Affiliated Hospital of Xi’an Jiaotong University, Shaanxi 710061, China; Department of Infectious Diseases, the First Affiliated Hospital of Xi’an Jiaotong University, Shaanxi 710061, China; Key Laboratory of Surgical Critical Care and Life Support, Biobank, Centre for Biobank and Advanced Medical Research of Shaanxi Province, the First Affiliated Hospital of Xi’an Jiaotong University, Shaanxi 710061, China; Department of Infectious Diseases, the First Affiliated Hospital of Xi’an Jiaotong University, Shaanxi 710061, China; Key Laboratory of Surgical Critical Care and Life Support, Biobank, Centre for Biobank and Advanced Medical Research of Shaanxi Province, the First Affiliated Hospital of Xi’an Jiaotong University, Shaanxi 710061, China; Department of Infectious Diseases, the First Affiliated Hospital of Xi’an Jiaotong University, Shaanxi 710061, China; Key Laboratory of Surgical Critical Care and Life Support, Biobank, Centre for Biobank and Advanced Medical Research of Shaanxi Province, the First Affiliated Hospital of Xi’an Jiaotong University, Shaanxi 710061, China; Department of Biochemistry and Molecular Biology, Taikang Medical School (School of Basic Medical Sciences), Wuhan University, 430072, China; BioCrystallisation Lab, Chemical Engineering Department, Loughborough University, Loughborough LE11 3TU, United Kingdom; Department of Life Sciences, Imperial College London, London, SW7 2AZ, United Kingdom; Department of Life Sciences, Imperial College London, London, SW7 2AZ, United Kingdom; State Key Laboratory of Virology and Biosafety, Wuhan Institute of Virology, Chinese Academy of Sciences, Wuhan, Hubei 430071, China; Department of Pathogen Biology, School of Basic Medicine, Tongji Medical College and State Key Laboratory for Diagnosis and Treatment of Severe Zoonotic Infectious Diseases, Huazhong University of Science and Technology, 13 Hangkong Road, Wuhan 430030, China; Department of Infectious Diseases, the First Affiliated Hospital of Xi’an Jiaotong University, Shaanxi 710061, China; Key Laboratory of Surgical Critical Care and Life Support, Biobank, Centre for Biobank and Advanced Medical Research of Shaanxi Province, the First Affiliated Hospital of Xi’an Jiaotong University, Shaanxi 710061, China; Department of Life Sciences, Imperial College London, London, SW7 2AZ, United Kingdom

## Abstract

The arms race between phages and bacteria is dynamic and ongoing, with both continuously acquiring new strategies to outcompete each other during co-evolution. Here, we report bacteriophage T4 exonuclease DexA and an uncharacterized *Escherichia coli* exonuclease as a rare pair of attack and defense duo arising from the same mechanism. DexA, highly conserved among phages, has two well-characterized biological roles: host DNA scavenging and intron homing. Unmodified DNA is the substrate during host DNA scavenging, whereas cleavage of 5hmC (5-hydroxymethylcytosine)-modified phage DNA is required for intron homing. We reveal a temperature-driven quaternary fold switch between DexA dimer and tetramer that facilitates cleavage of distinct DNA forms, namely 5hmC-modified phage DNA and unmodified host DNA. As a countermeasure, bacteria produce DexA variants for defense against phage that only targets 5hmC-modified DNA. Thus, both phages and bacteria compete using HmC-Recognizing EXonuclease strategies (designated as HREX).

## Graphical Abstract

**Graphical Abstract:**
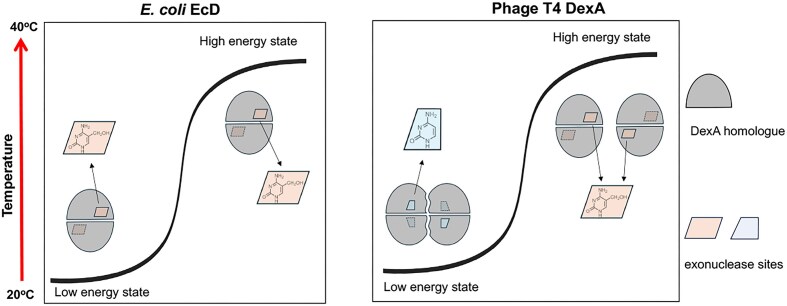

## Introduction

Phages are viruses that infect bacteria and are incredibly numerous, outnumbering bacteria by a ratio of ∼10:1 [1]. A lytic phage attacks its bacterial host by attaching to the surface of the bacterium and injecting its DNA into the host. It then quickly repurposes the bacterial metabolic processes to produce its own progeny, which will eventually burst out of the bacterial cell. In responding to phage invasions, bacteria have developed a wide range of antiphage defense systems that can provide partial or full protection against phages [2]. During the invasion process, phage DNA and proteins may be detected as foreign by the bacteria, triggering immune responses. Meanwhile, bacteria also monitor the integrity of their own cellular machinery for the activation of defenses. Over a hundred defense systems have been known so far, consisting of either single genes or groups of genes. Analyzed by DefenseFinder [3], restriction–modification (RM) and CRISPR–Cas are the most common and are found to be encoded by >80% of the bacterial genomes, as the detection of invading nucleic acids is one of the earliest steps that bacteria can launch a defense. In the RM systems, DNA modifications are normally introduced on specific motifs in the host genome and bacterial restriction endonucleases (REase) recognize the same sequence in invading nucleic acids that lack those modifications [4]. However, REase of the type IV RM system can also recognize and cleave the specific DNA sequence with modifications on the phage [5]. While sequence recognition and endonucleolytic activity are the common features shared by the RM systems, exonucleolytic enzyme and nonspecific sequence recognition systems are rarely described as antiphage strategies.

Bacteriophage T4 is one of the most studied lytic phages that infects *Escherichia coli* [6]. The T4 genome encodes about 300 gene products, coding many nonessential and functionally redundant gene products to cope with different and stressful environments. A notable feature of T4 DNA is that all cytosines are replaced by 5-hydroxymethylcytosine (5hmC), and is subjected to further modification by hydroxymethyl glucosylation (glc-HMC) [6, 7]. During infection, T4 degrades host genome to meet its requirement for a large nucleotide pool to support rapid DNA replication [8]. And the difference in 5hmC-modified T4 DNA and unmodified *E. coli* DNA provides multiple advantages, including the degradation of host DNA, but not its own, using a phage-encoded nuclease [9]. As a T4 encoded gene product, DexA (also referred to ExoD in the literature) was first identified as a 3′–5′ exonuclease because T4 strains carrying mutations in *dexA* lost exonuclease activity [10]. While T4 exhibits redundancy in its DNA replication and recombination systems to adapt to various environments [6], DexA is essential for successful replication in *E. coli* strains with mutations in the *optA* gene. The *Dgt* gene, which is affected by *optA* mutations, encodes a deoxyguanosine triphosphate triphosphohydrolase responsible for hydrolyzing dGTP and releasing triphosphate. In this context, DexA plays a critical role in T4 replication within *E. coli* hosts carrying *optA* mutations by providing a mononucleotide pool for phage replication through the cleavage of host DNA [11]. Evidently, no T4 DNA is synthesized in the *optA* mutant that is infected by phage carrying a *dexA* deletion [12]. The T4 genome also undergoes intron homing, a gene mobility process in which a self-splicing group I intron, guided by its encoded homing endonuclease, inserts into an intron-less allele through site-specific DNA cleavage and homologous recombination [13]. During this process, initiated by its intron endonuclease I-TevI, both DexA and the proofreading domain of T4 DNA polymerase (43Exo of Gp43) contribute to the homing event due to their 3′–5′ exonuclease activity with 5hmC-modified T4 DNA as the substrate [14]. Unlike most other nucleases, DexA can target both unmodified *E. coli* DNA and modified T4 DNA substrates. The molecular mechanisms that allow DexA to accommodate two different substrates in two distinct biological roles remain unclear.

Here, we report DexA and a previously uncharacterized *E. coli* exonuclease, which we have termed EcD, are phage attack and bacterial defense proteins, respectively, which utilize the same discrimination strategy for 5hmC-modified DNA. Our biochemical and structural studies reveal that DexA is a dual-function exonuclease that undergoes a temperature-dependent quaternary structure change, a structural transformation in the assembly of monomers into a functional multimeric complex. DexA switches between dimeric and tetrameric conformers at two physiologically relevant temperatures, 25°C and 37°C. Cryo-electron microscopy (cryo-EM) and crystallography data suggest that it exists in a dimer–tetramer equilibrium, with the tetramer state populated at 25°C, while the dimer mostly present at 37°C. We then demonstrated that the state with substantial tetramer present is responsible for scavenging host *E. coli* DNA at 25°C, while the dimer state at 37°C is utilized for phage intron homing using T4 DNA as the substrate. Strikingly, we found that bacteria also encode a DexA homologue, EcD, that resembles the dimer state and only cleaves 5hmC-modified DNA. *Escherichia coli* carrying a plasmid-borne EcD shows resistance to T4 even without induction. Our findings reveal that while T4 and other DexA-containing phages take advantage of the difference in DNA modification between host and phage, bacteria counterstrike with an exonuclease that solely recognizes phage DNA based on the same modification recognition, i.e. HmC-Recognizing EXonuclease (we termed HREX).

## Materials and methods

### Plasmid construction and mutagenesis

The DexA (*dexA*) gene was amplified from T4 genome through polymerase chain reaction (PCR) and purified using gel extraction. The purified DNA fragment was cloned into respective vectors based on Gibson assembly. The primers for site-directed mutagenesis were designed using the NEB online design software, NEBaseChanger. Mutations were achieved by using back-to-back PCR amplifications with forward primers bearing the target mutation. The resulting plasmids were transformed into *E. coli* DH5α cells for amplification. All primers used in this study are listed in Supplementary Table S1.

### Bacterial growth attenuation assays

The sequenced pET vectors were electroporated into *E. coli* BL21(DE3) competent cells. BL21(DE3) containing an empty circular pET46 vector was used as the negative control. The colonies were inoculated into 2 ml LB medium containing 5 μg/ml ampicillin and cultured overnight at 37°C and 220 rpm. The culture was then diluted to a fresh medium containing 5 μg/ml of ampicillin at a ratio of 1:100. For the induced group, 1 μM of isopropyl-β-d-thiogalactopyranoside (IPTG) was added. Two hundred microliters of culture was transferred to a 96-well microplate, and OD_600_ was measured by a Neo-2 porous plate reader (Bio Tek) at 25°C and 37°C, respectively. Three biological and technical replicates were performed for each growth assay.

### Protein expression and purification

The pET46 vector bearing *dexA* gene or its mutants was transformed into *E. coli* BL21(DE3) for recombination protein expression with a 6× His-tag at N-terminal. The cells were grown at 37°C till OD_600_ ∼ 0.6 before being induced using 1 mM of IPTG in LB media or M9 media containing ^15^N-labeled NH_4_Cl as the sole nitrogen source. The induced cells were incubated at 18°C for 16–18 h before harvesting. The cell culture was harvested by centrifugation at 5422 × *g* for 10 min at 4°C, resuspended in 50 ml of binding buffer (50 mM NaH_2_PO_4_, 300 mM NaCl, 10 mM imidazole, pH 8.0), and lysed by sonication. The lysate was then centrifuged at 47 850 × *g* for 30 min at 4°C to harvest the soluble fraction. The supernatant was subjected with standard His-tag protein purification using a benchtop Ni-NTA column. After purification, the protein was immediately dialyzed overnight at 4°C with storage buffer (50 mM Tris–HCl, 150 mM NaCl, 0.2 mM tris(2-carboxyethyl) phosphine, pH 7.5). Finally, the protein was concentrated to a volume of 2 ml, filtered through a 0.22-μm filter, and subjected to size exclusion chromatography (SEC) using a Superdex 75 HiLoad 16/600 column in the ÄKTA Pure system (GE Healthcare Life Sciences) for further purification.

### Phage infection growth curve

For the bacterial growth inhibition assay, *E. coli* BL12(DE3) cells bearing empty pET46 vector, or plasmid-borne DexA or EcD were grown overnight in LB medium with ampicillin and 1% glucose, diluted to mid-log phase, and mixed with phages at desired Multiplicity of infection (MOI) by diluting from a high-titer phage T4 stock (10^11^ PFU/ml). For the induction group, 0.3 μM of IPTG was added to the culture. Two hundred microliters of culture was transferred to a 96-well microplate, and OD_600_ was measured by a Neo-2 porous plate reader (Bio Tek) at 37°C. The concentration of *E. coli* was determined by the standard plate count method, and the plaque-forming units (PFU) of T4 were calculated using the standard plaque assay. The MOI is the ratio of T4 to *E. coli* cells. Three biological and technical replicates were performed for each growth assay.

### Ion exchange chromatography

The SEC-purified protein was subjected to further separation using an anion exchange column HiTrap Q-FF (GE Healthcare Life Sciences) and ÄKTA Pure system. The column was first washed with 2 column volumes of buffer A (50 mM Tris–HCl, pH 7.5), and elution was initiated by using 20 column volumes of elution buffer B (50 mM Tris, 1.5 M NaCl, pH 7.5) at a linear gradient.

### Exonucleolytic activity assay on ssDNA and dsDNA

The purified samples were serially diluted into the desired concentrations and mixed with 100 μM single-stranded DNA (ssDNA; oligo1) or double-stranded DNA (dsDNA; assembled by using oligo1 and oligo2). The fragment 2 was amplified by standard PCR using the sequence and primer listed in Supplementary Table S1. And the 5hmC-modified fragment 2 was using 5-hydroxymethyl-deoxycytidine triphosphate (5-hydroxymethyl-dCTP, Jena Bioscience) instead of dCTP and Zymo Taq DNA Polymerase as described previously [15]. The final volume of the reaction mixture was 10 μl in a reaction buffer (50 mM Tris–HCl, 10 mM MgCl_2_, pH 7.5) with a constant DNA concentration of 10 μM. The molar ratios of protein to oligonucleotide are listed in figure captions. After incubation at relevant temperature for 30 min (except for Supplementary Fig. S1A, which were incubated for 3 h), the nucleolytic reaction was terminated by heating samples at 95°C for 3 min. For the experiment using ssDNA or dsDNA as substrates, the deactivated reaction mixtures were separated on Novex TBE Gels (Thermo Fisher) at 120 V for 30 min.

### Exonucleolytic activity assay using T4 genome as substrate

For assays using T4 DNA as the substrate, T4 genome was exacted from cell-free high-titer phage lysate using the M13 DNA Mini Kit (Omega Bio-tek). The reaction was incubation at relevant temperatures for 3 h (except for Supplementary Fig. S1B, which were incubated for 10 h) before deactivation and electrophoresis using 1% agarose gels at 120 V for 60 min. The molar ratios of protein to T4 genome were as follows: 0.5:1, 1:1, 1:2, and 1:4, unless indicated.

### X-ray crystallography analysis and structure determination of protein crystals

The purified proteins were concentrated to 20 mg/ml and crystals were grown using sitting drop vapor diffusion. One microliter of DexA was mixed with 1 μl of precipitant solution containing 0.02 M calcium chloride dihydrate, 0.1 M sodium acetate trihydrate (pH 5.3), and 25% (v/v) (±)-2-methyl-2,4-pentanediol, while 1 μl of DexA^S87A/D90A^ was mixed with 1 μl of precipitant solution containing 0.2 M magnesium chloride hexahydrate, 0.15 M HEPES sodium (pH 7.5), and 25% (v/v) polyethylene glycol 400. Crystals for both samples appeared after 24 h. The DexA and DexA^S87A/D90A^ crystals were transferred to precipitant solution containing 25% glycerol prior to flash-freezing in liquid nitrogen before subjected to X-ray diffraction analysis. The data collections were done at beamline BL17U1 of Shanghai Synchrotron Radiation Facility. A total of 180 frames were recorded with a 1° oscillation angle and 0.2 s exposure time per frame. The data were processed for indexing, integration, and scaling using the HKL3000 program package. The protein structure was determined and refined using the Phenix suite program. A homologous structure-based phaser was employed as a search model for initial phase determination, followed by model building using Coot. The resulting model was further refined using Phenix.refine, and the quality of the structure, including Ramachandran plots and residue moieties, was assessed through the evaluation of electron density maps. The data refinements and statistics are listed in Supplementary Table S2.

### SEC–MALS

The SEC–MALS (multi-angle light scattering with size-exclusion chromatography) setup is temperature controlled, with a range from 25°C to 90°C, using a sealed temperature controller that houses both the column and detector. A 100 μl protein sample was injected at a flow rate of 0.5 ml/min into a Superdex 75 10/300 GL size-exclusion column (GE Healthcare), which was connected to an Agilent HPLC 1100 system (including a degasser and pump) in line with a multi-angle laser light scattering detector (miniDAWN TREOS, Wyatt Technology, Santa Barbara, CA) and a relative refractive index interferometer (Optilab T-rEX refractometer, Wyatt Technology). ASTRA 6.1 software was utilized for data collection and analysis. At 25°C, the solvent refractive index was defined as 1.331, and the solvent viscosity was defined as 0.8945 cP, while at 37°C, the solvent refractive index was defined as 1.329, and the solvent viscosity was defined as 0.6917 cP, with a refractive index increment value (dn/dc) defined as 0.185 ml/g for both temperatures. The errors for the calculated molecular weights (MWs) are within 4%.

### Cryo-EM sample preparation, and data collection and processing

To investigate the structural differences of the ExoD protein at various temperatures, purified protein samples were incubated at 25°C and 37°C for 1 h. For each condition, 3.5 μl of the sample was applied to a glow-discharged 300-mesh copper (Cu) R1.2/1.3 holey carbon grids (Quantifoil). The grids were blotted for 2 s at 4°C with a blotting force of 5 before being plunge-frozen in liquid ethane using an FEI Vitrobot Mark IV (Thermo Fisher Scientific). Both samples incubated at 25°C and 37°C were briefly exposed to 4°C for a few seconds during transfer to the grids. The brief exposure should not significantly impact the binding states of the proteins, as supported by the pronounced differences observed in tetramer formation at the two incubation temperatures.

Data acquisition was conducted using a CRYO ARM 300 electron microscope (JEOL, Japan) at an operating voltage of 300 kV and equipped with a K3 direct electron detector (Gatan, USA). Cryo-EM images were captured in super-resolution mode, with a physical pixel size of 0.95 Å. A total of 3650 movie series were collected for the sample incubated at 25°C, and 3301 movie series for the 37°C sample, each at a frame rate of 40 fps and a total electron dose of 40 e^−^/Å^2^.

Data processing was consistently performed for both sets using cryoSPARC [16], involving patch motion correction, patch CTF estimation, 2D classification, *ab initio* reconstruction, heterogeneous refinement, and nonuniform refinement. The crystal structure of the protein dimer was then fitted into the cryo-EM maps using ChimeraX [17].

### Molecular dynamic simulation

Molecular dynamic (MD) simulations were meticulously conducted to assess the stability of the protein complex and the binding property of the substrate DNA. The initial files were prepared before modeling and simulations using CHARMM-GUI [18] solution builder, allowing for the comprehensive generation of the MD input. The force fields employed were CHARMM36m [19], ions (0.15 M KCl), and the TIP3 model water molecules. For the stability simulation, we ran the energy minimization for the whole system first. Next, the simulations were carried out at 25°C or 37°C using a canonical NVT ensemble, where the temperature was controlled by the Nosé–Hoover thermostat [20]. Then, NPT ensemble was performed in production phase, where the target pressure and temperature were 1 atm and at 25°C  or 37°C, respectively. Default tether restraints from Lammps were applied to the system. The subsequent MD production employing a 2-fs time step for 1 ns. For the substrate DNA binding simulation, the substrate was manually fitted in the dimer structure according to the complex structure reported earlier (PDB ID: 1J54) using the same simulation parameters as above. The comprehensive analysis of simulation outputs and structural features was carried out utilizing Lammps [21], ChimeraX, Pymol [22], and VMD [23].

### NMR spectroscopy

All nuclear magnetic resonance (NMR) experiments were recorded on a Bruker 800 MHz (Avance III) spectrometer at 298 or 310 K. The ^15^N–^1^H HSQC spectra were recorded at a protein concentration of 0.5 mM. The sample was in a buffer containing 50 mM Tris, 150 mM NaCl, and 0.2 mM tris (2-carboxyethyl) phosphine (pH 7.5).

### Phage hosts and bacteria clustering and network analysis

The DexA sequence was aligned against the NCBI nr database using BLASTp with an *E*-value threshold of 0.001, identifying 381 unambiguous phage and 24 534 bacterial protein sequences. Phage sequences were extracted and clustered using CD-HIT at an 85% identity threshold, resulting in 112 clusters. For bacterial sequences, only those aligning with the RefSeq database were retained, resulting in a dataset of 8238 sequences, including 5615 from *Escherichia* and 1007 from *Klebsiella*. These sequences were respectively dereplicated using CD-HIT at a 99% identity threshold, producing 1217 *Escherichia* and 363 *Klebsiella* sequences. The resulting sequences were combined with 1616 sequences from other genera and clustered at an 85% identity threshold, yielding 350 clusters. Network graphs were generated using Gephi to visualize the relationships between sequences within each cluster. The phages and bacteria are listed in Supplementary Table S3.

### Phage plaque assay

The plaque assay was performed using a soft agar overlay technique as previously described [24]. For the plaque assay, bacterial cells were grown to an OD_600_ of ∼0.3 at 37°C. Soft agar (0.5% agar, 1 mM MgCl_2_) was boiled and allowed to cool to 42°C. A 100 μl aliquot of bacterial cells was added to 10 ml of soft agar, poured into a Petri dish plate, and solidified with a closed lid. The phage lysate was serially diluted in LB media from 1 × 10^−1^ to 1 × 10^−6^. A 5 μl volume of each phage lysate dilution was spotted onto the soft agar plate. In the induced control and EcD groups, 0.3 μM IPTG was also included in the double-layered agar. Overnight, plaques became visible and images were taken.

## Results

### DexA has a temperature-dependent substrate preference

**Figure 1.**
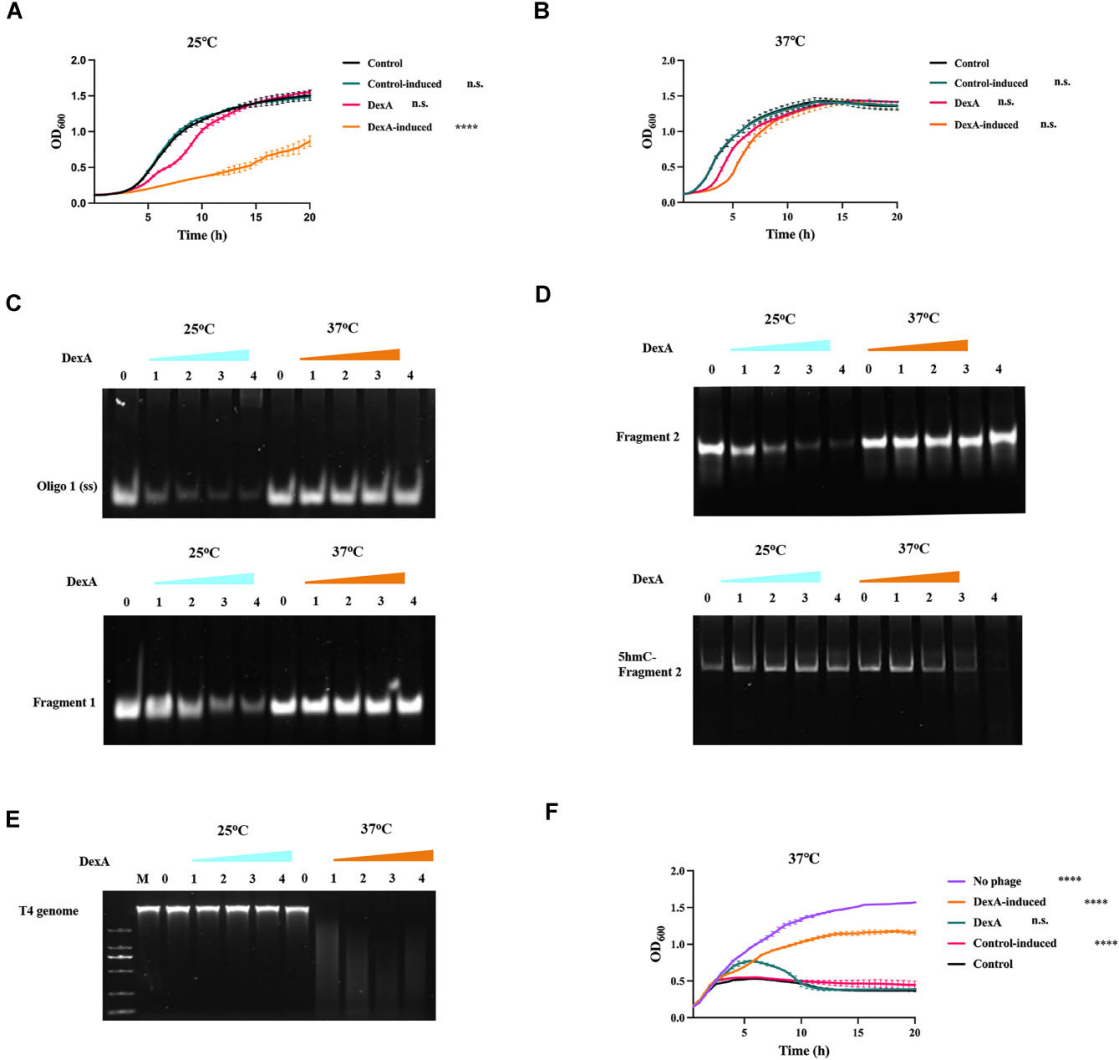
DexA has a temperature-dependent substrate preference. (**A**,**B**) The growth curves of *E. coli* overexpressing DexA at 25°C and 37°C, respectively. The control bears an empty circular vector. Error bars show SEM; n.s., not significant (*P*> .05), ^****^P< .0001; two-way ANOVA, *n* = 3. (**C**) Top: The TBE gel showing the exonuclease activity assay of DexA using ssDNA (oligo 1) as the substrate; bottom: the TBE gel showing the exonuclease activity assay of DexA using fragment 1 as the substrate. The concentrations of oligo 1 and fragment 1 were both 10 μM, while the concentrations for DexA were 5, 10, 15, and 20 μM, corresponding to labels 1–4, respectively. (**D**) Top: The TBE gel showing the exonuclease activity assay of DexA using fragment 2 (100 bp) as the substrate; bottom: the TBE gel showing the exonuclease activity assay of DexA using 5hmC–fragment 2 as the substrate. The concentration of unmodified fragment 2 was 10 μM, while the concentrations for DexA were 5, 10, 15, and 20 μM, corresponding to labels 1–4, respectively. And the concentration for 5hmC–fragment 2 was 0.5 μM, while the concentrations for DexA were 0.5, 1, 2, and 4 μM, corresponding to labels 1–4, respectively. (**E**) The agarose gel showing the exonuclease activity assay of DexA using T4 genome as the substrate. The concentration of T4 genome was 200 ng/μl (∼3 × 10^−3^ μM), while the concentrations for DexA were 1, 2, 4, and 8 μM, corresponding to labels 1–4, respectively. The reaction temperature is labeled above the gels. (**F**) The growth curves of T4 infecting *E. coli* with plasmid-borne DexA at 37°C. The control is an empty circular plasmid and T4 phage was diluted from a high-titer stock to achieve an MOI of 10^−3^. Error bars show SEM; n.s., not significant (*P* > .05), ^****^P < .0001; two-way ANOVA, *n* = 3.

To study the mechanism by which it distinguishes host and T4 DNA, we first recombinantly expressed DexA in its native host *E. coli*. Interestingly, overexpressing DexA exhibited toxicity to *E. coli* at 25°C (Fig. 1A), yet bacterial growth was much less affected at 37°C (Fig. 1B). To check whether the growth attenuation originates from temperature-dependent exonuclease activities of DexA, we assessed the enzymatic activity of recombinant DexA *in vitro*. Using ssDNA (oligo 1) and dsDNA fragment 1 (formed by using complementary oligo2 with oligo1) as the substrates (oligos listed in Supplementary Table S1), DexA exhibited a strong nucleolytic activity in a dose-dependent manner using ssDNA as its substrate at 25°C. Strikingly, no noticeable nucleolytic activity was observed at 37°C (Fig. 1C). It also showed a similar temperature-dependent nucleolytic activity against dsDNA, yet weaker, which agrees with its documented preference for ssDNA over dsDNA [12]. The fact that DexA has higher exonucleolytic activity at lower temperature is puzzling; however, it is consistent with the scavenger role of DexA during stress, i.e. low temperature. We therefore speculated that intron homing may be its role at optimal temperature, as T4 is known to reduce the self-splicing of group I introns under unfavorable conditions [25]. Using a 100-bp fragment 2 with or without 5hmC modification, we demonstrated that DexA prefers plain DNA at 25°C but 5hmC-modified DNA at 37°C as substrate (Fig. 1D). Additional temperatures (20°C, 30°C, and 40°C) were tested (Supplementary Fig. S1C and D). The results demonstrate a temperature-dependent gradient in DexA activity, with 37°C likely representing the upper threshold for activity on unmodified DNA, and 30°C serving as the lower threshold for activity on 5hmC-DNA at the concentrations and temperatures tested. As the cytosines in the T4 genome are subject to further glucosylation in addition to the 5hmC modification, we used purified T4 genome to show that DexA indeed cleaves the 5hmC-modified T4 genome at 37°C, but not at 25°C (Fig. 1E). However, a significant enzyme-to-DNA ratio (3 × 10^4^) is required to completely cleave the T4 genome, suggesting that the exonucleolytic activity of DexA against its own DNA is limited. While the temperature-depend substrate preference is strong, it is not fully exclusive. By extending the reaction time, DexA showed some limited nucleolytic activity against T4 genome at 25°C, and fragment 2 at 37°C (Supplementary Fig. S1A and B). This is also supported by the postponed growth in DexA-bearing *E. coli* at 37°C (Fig. 1B). Based on its substrate preference *in vitro*, DexA-overexpressing *E. coli* cells should be granted T4 immunity or at least partial protection at 37°C. A T4 infection assay confirms this hypothesis, showing that DexA confers *E. coli* protection against the T4 at 37°C (Fig. 1F). These *in vitro* and *invivo* studies reveal that DexA has temperature-dependent exonucleolytic activity that switches its substrate preference between *E. coli* and T4 DNA, consistent with its roles in DNA scavenging at <30°C, when it was discovered as an essential nuclease for *E. coli* carrying *optA* mutations [12, 26], and intron homing at optimal temperature conditions.

### DexA undergoes temperature-driven quaternary structure change

**Figure 2.**
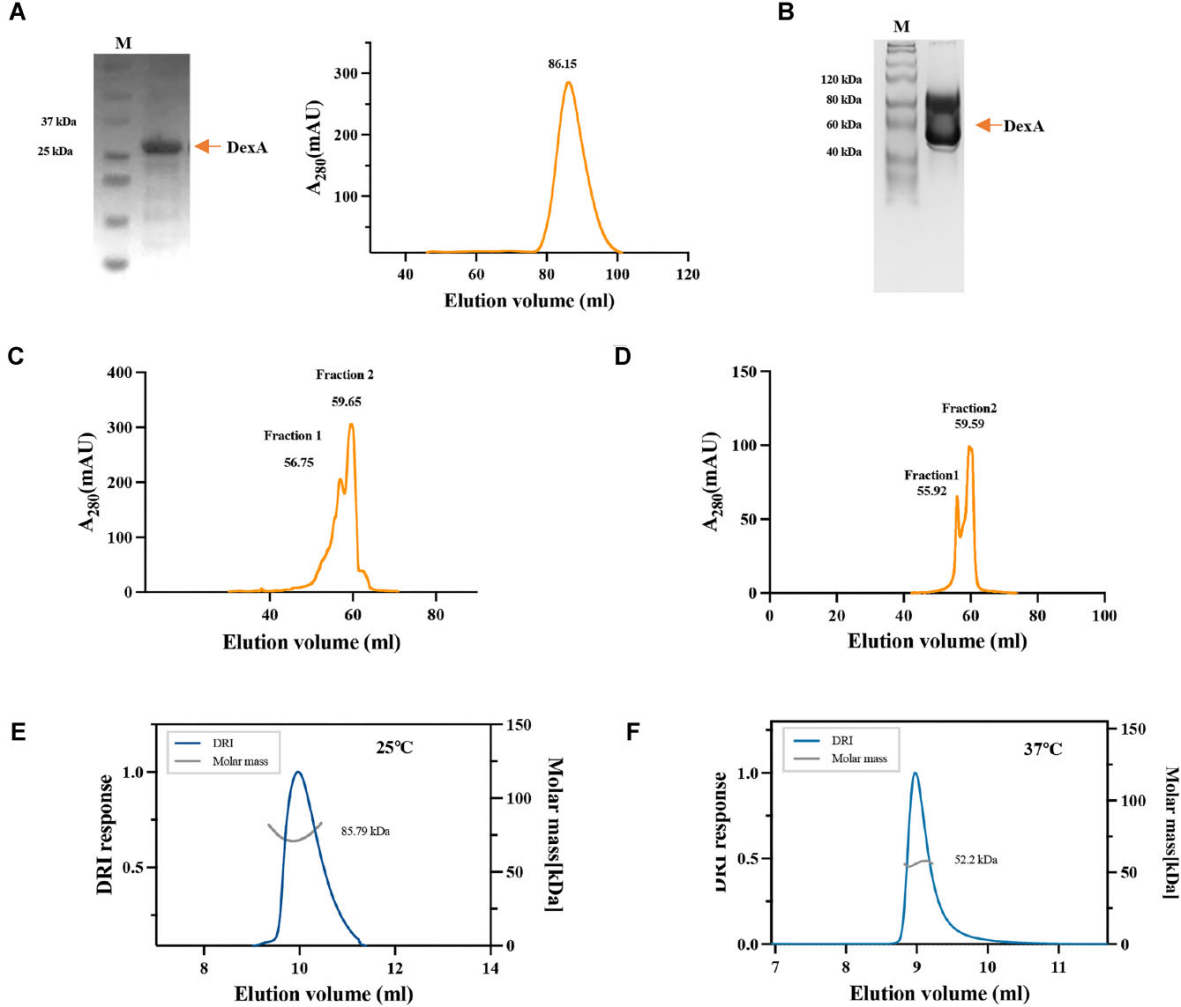
DexA undergoes temperature-driven quaternary structure change. (**A**) The SDS–PAGE image showing the recombination expression of DexA and the size exclusion chromatographic profile of the purified recombinant DexA using a Superdex 200 HiLoad 26/600 column. (**B**) The native gel electrophoresis of the purified DexA after the SEC. (**C**) The ion exchange chromatographic profile of DexA purified in panel (A) using a Mono Q column: fraction 1 represents the peak at 56.8 ml and fraction 2 represents the peak at 59.7 ml. (**D**) The ion exchange chromatographic profile of fraction 2 purified in panel (C) using a Mono Q column: fraction 1 represents the peak at 55.9 ml and fraction 2 represents the peak at 59.6 ml. All experiments including protein purification, chromatography, nuclease activity assay, and electrophoresis were performed at room temperature. (**E**,**F**) The SEC–MALS profiles of DexA at 25°C and 37°C, respectively.

To explain the substrate-switching mechanism of DexA, we first speculated that DexA may have two forms: one active at low temperature and the other at high temperature. To verify this, we attempted to separate the two forms using chromatography. While the recombinant protein appeared as a single band at around 26 kDa on an SDS–PAGE gel, the estimated MW is around 60 kDa based on its elution volume (86.2 ml) in SEC on a Superdex 200 column (Fig. 2A). This suggests that DexA is not monomeric (26.0 kDa per monomer) and likely to be at least dimeric in PBS (phosphate buffered saline) buffer at room temperature, 22°C (Fig. 1A). This multimerized form of DexA is not unexpected, as *E. coli* exonucleases generally adopt different quaternary structural arrangements, including monomeric, dimeric, and larger complexes [27]. The sample was then subjected to native gel electrophoresis to verify whether DexA exists as a higher order multimer. Interestingly, two major bands at around 100 and 50 kDa, suggested that DexA exists in a mixture of dimeric and tetrameric forms (Fig. 2B).The presence of a smear between these bands, coupled with the single peak observed in SEC, suggests dynamic equilibrium and exchange between the two multimeric states. The sample was then subjected to ion exchange chromatography (IEX), which showed two peaks eluting at 56.8 and 59.7 ml suggesting two conformations, with the latter being the dominant fraction (Fig. 2C). Although it was not possible to fully separate the two fractions, we collected the dominant fraction (fraction 2) and subjected it to another round of IEX. The chromatogram profile is similar to the first IEX, suggesting that the two fractions exist in equilibrium (Fig. 2D). In summary, SEC, native gel electrophoresis, and IEX all suggest that DexA exists in a dimer–tetramer equilibrium at room temperature.

We next examined whether the temperature could affect the equilibrium between dimeric and tetrameric DexA. Using SEC–MALS, we determined the absolute MW of DexA under 25°C and 37°C. The results show that DexA has an MW of 52.2 kDa at 37°C, which is equivalent to a dimer, and an increased MW of 85.8 kDa at 25°C, indicating the likely formation of a tetramer population (Fig. 2E and F). The results suggest DexA undergoes temperature-driven quaternary structure change between dimer and tetramer, with the dimeric form predominant at higher temperatures.

### X-ray crystallography and cryo-EM characterization of DexA

**Figure 3.**
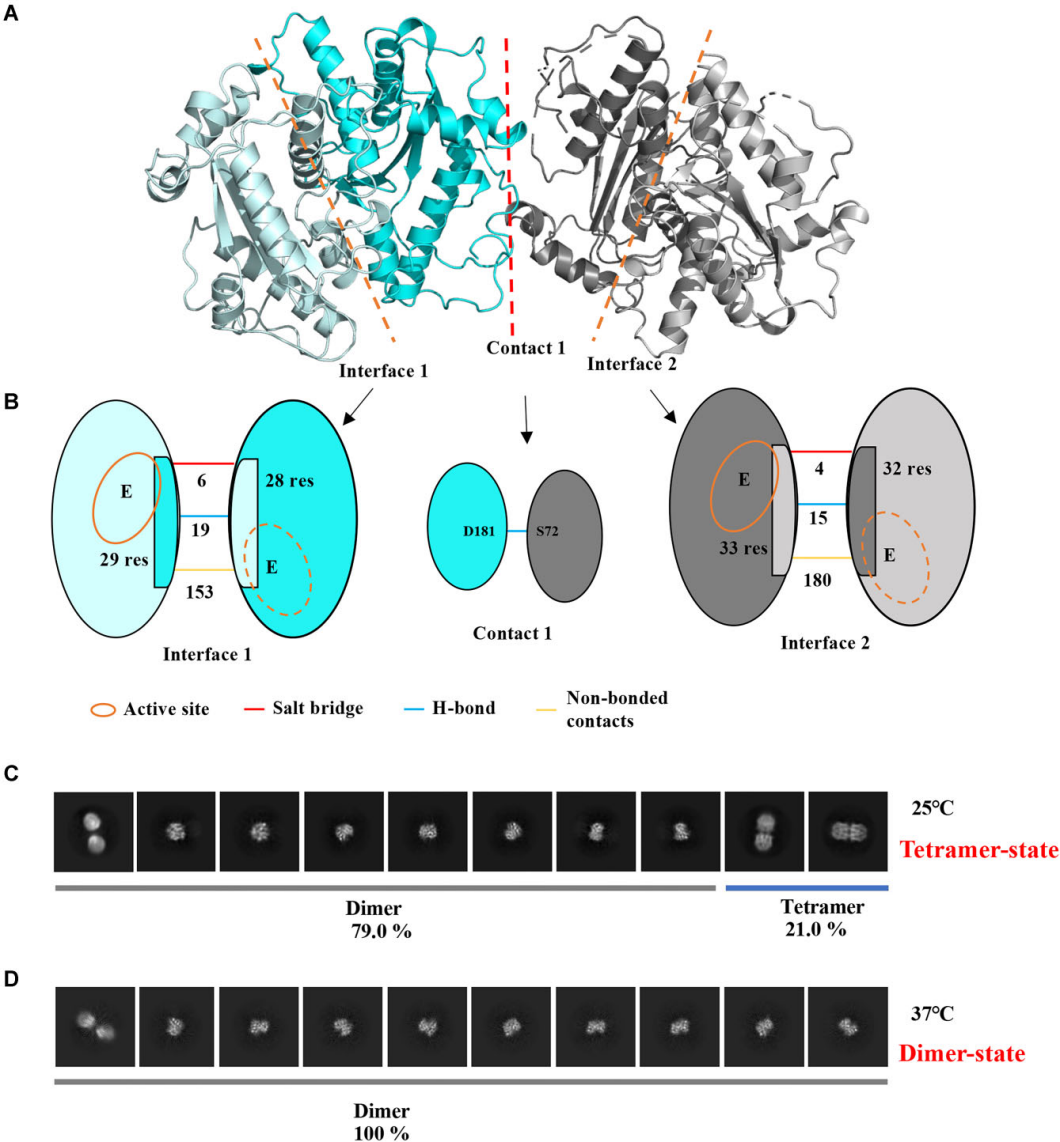
DexA undergoes temperature-dependent quaternary structure change. (**A**) The cartoon representation of the 2.88 Å dimeric structures of DexA complexes. The two coexisting isomers in the asymmetric unit are colored in two-tone gray for isomer 1 and two-tone cyan for isomer 2. (**B**) The schematic illustration of the interactions at interfaces 1 and 2. The number of hydrogen bonds, salt bridges, and other nonbonded contacts, as well as the residues involved in the interfaces, are labeled as indicated. A single hydrogen bond is formed between D181 and S72 at contact 1. (**C**) 2D classification of DexA at 25°C: 79% in dimeric form and 21.0% in tetrameric form (tetramer state). (**D**) 2D classification of DexA at 37°C shows it exists solely in dimeric form (dimer state).

To understand more on the quaternary structure change, we solved the DexA structure by X-ray crystallography. Reproducible and well-diffracting crystals were obtained at pH 4.6 for the native full-length DexA. Molecular replacements were performed with PHASER using the search model of DexA predicted by AlphaFold2 [28, 29]. DexA exists as a tetramer in the asymmetric unit, which is formed by two loosely packed dimers. Notably, the structural arrangements of the two dimers are different. Analyzed by PDBsum [30], dimer interface 1 between the two subunits shown is 1576 Å^2^ and involves 6 salt bridges, 157 nonbonded contacts, and 18 hydrogen bonds, whereas dimer interface 2 is 1546 Å^2^ and involves 4 salt bridges, 180 nonbonded contacts, and 17 hydrogen bonds (Fig. 3C and Supplementary Fig. S2B). The contact between the dimers (contact 1) is much smaller, with an interface of 264 Å^2^ and involves 29 nonbonded contacts and 1 hydrogen bond (Supplementary Fig. S2C). Therefore, the dimer interfaces 1 and 2 are similar but not identical, and the tetramer is formed by joining two dimers mainly via a hydrogen bond between S72 and D181. The existence of slightly different conformations for the two dimers suggests a degree of conformational flexibility. In summary, DexA adapts two slightly different dimeric conformations in the tetrameric asymmetric unit.

To examine the multimeric state of DexA further, we utilized single-particle cryo-EM to capture the states at two temperatures, 25°C and 37°C, at a near-physiological concentration of 5 μM [31] (Supplementary Figs S3A–E and S4A–D). Although it was not possible to determine a high-resolution structure *ab initio*, we could use 2D classifications to estimate the percentage of different conformers. At 25°C, the exonuclease has two forms: dimer and tetramer. Based on 2D classification, 79.0% of DexA molecules are dimeric and 21.0% are tetrameric (Fig. 3C and Supplementary Fig. S3C). This composition is consistent with IEX profiles of the sample at room temperature (Fig. 2C and D). At 37°C, DexA only exists in the dimeric form (Fig. 3D and Supplementary Fig. S3D). By lowering the temperature to 4°C, we observed the formation of higher order multimers (Supplementary Fig. S3E). 2D classification suggests that the percentage of dimer drops to 42.5%, while the tetramer and multimers are 21.9% and 35.6%, respectively. The EM results suggest that dimeric and tetrameric forms of DexA can coexist at biological relevant temperatures and multimerization to tetramer and beyond is promoted at lower temperatures.

### Crystal structure and characterization of the DexA^S87A/D90A^ mutant

**Figure 4.**
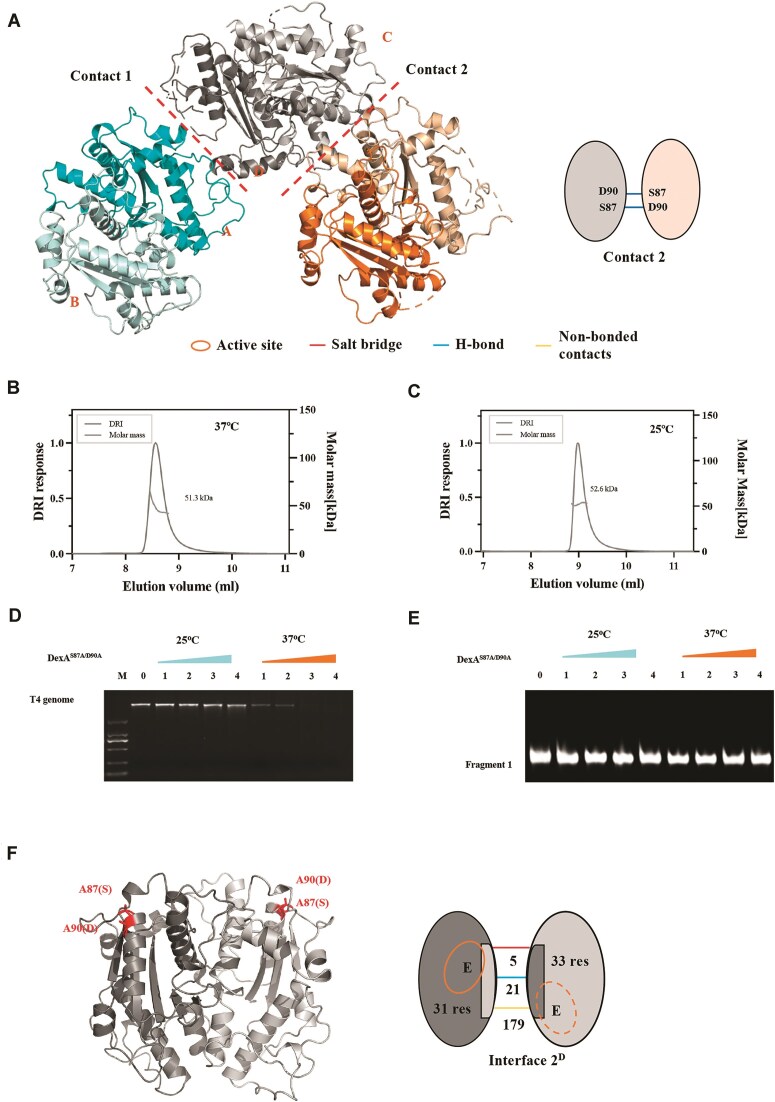
Structural and functional characterization of DexA^S87A/D90A^. (**A**) The cartoon representation of the DexA structure that includes the extended crystal contacts. The two mutual hydrogen bonds are formed between D90 and S87 at contact 2. The dimer in orange is structurally identical to the dimer in gray, but different from blue. The number of hydrogen bonds, salt bridges, and other nonbonded contacts, as well as the residues involved in the interfaces, are labeled as indicated. (**B**,**C**) The SEC–MALS profiles of DexA at 25°C and 37°C, respectively. (**D**) The agarose gel showing the exonuclease activity assay of DexA^S87A/D90A^ using T4 genome as the substrate. The concentration of T4 genome was 200 ng/μl (∼3 × 10^−3^ μM), while the concentrations for DexA^S87A/D90A^ were 0.25, 0.5, 1, and 2 μM, corresponding to labels 1–4, respectively. The reaction temperature is labeled above the gels. (**E**) The TBE gel showing the exonuclease activity assay of DexA^S87A/D90A^ using fragment 1 as the substrate. The concentration of fragment 1 was 10 μM, while the concentrations for DexA were 5, 10, 15, and 20 μM, corresponding to labels 1–4, respectively. The reaction temperature is labeled above the gels. (**F**) The 2.38 Å crystal structure of DexA^S87A/D90A^ showing the dimeric structure of the mutant: 87A(S) and 90A(D) are labeled. The interactions involved in the interface 2^D^ are labeled on the right.

The tetramer is formed from two loosely packed dimers and the interface is dominated by a single hydrogen bond between S72 and D181 (contact 1). Further interactions between dimers can be found in the extended crystal network; i.e. another significant dimer–dimer interaction (contact 2) possesses two mutual hydrogen bonds between D90 and S87 (Fig. 4A). As the results show the presence of tetramer at 25°C, we sought to identify which contact facilitates tetramer formation in solution. To explore this, we first employed MD simulations of the crystal structure to study the dynamic nature of DexA at 25°C and 37°C (Supplementary Fig. S5A and Supplementary Movie S1 for contact 1, and Supplementary Fig. S5B and Supplementary Movie S2 for contact 2). During the simulation at 37°C, the tetramer was observed to separate at contact 1, but not contact 2; i.e. the complex breaks into two dimers at contact 1. This suggests the tetramer form at contact 2 interface is likely more stable in solution and perhaps functionally relevant. Furthermore, the two dimers remained bound together during the whole simulation at the low temperature of 25°C. Therefore, the MD simulations suggest that the contact 2 is more stable and likely involved in the tetramer formation.

As the crystal structure suggests contacts 1 and 2 could be important for the tetramer, we attempted to construct mutants that could break the tetramer formation. The mutant DexA^S87A/D90A^, which was designed to disrupt the tetramer via contact 2, appeared as a single peak during IEX (Supplementary Fig. S6A). The SEC–MALS result showed disruption of the mutant tetramer and that it remained dimeric at all temperatures (Fig. 4B and C). Nucleolytic assays confirmed that DexA^S87A/D90A^ loses the ability to cleave the unmodified DNA, but retains the ability to digest T4 genome (Fig. 4D and E). In contrast, the DexA^D181A/S72A^ mutant (contact 1) appears larger than the dimeric MW at 25°C, suggesting that the dimer–dimer interface remains intact (Supplementary Fig. S6B). These data are consistent with the predictions from MD simulations, showing that contact 2 is key for the tetramer formation.

Reproducible and well-diffracting crystals for the mutant were obtained under the same condition for wild-type DexA, and the structure was solved by molecular replacement as described before (Fig. 4F). The crystal structure of DexA^S87A/D90A^ is dimeric, suggesting that disrupting the dimer–dimer interface affects the quaternary (tetramer) structure but not the free dimeric unit wild type. The MD result showing that contact 2 does not separate at 37°C is not consistent with an absence of tetramers observed by EM at this temperature. While the simulation may not reflect the absolute temperature dependance of multimerization, it does show the trends in interface stability. Interestingly, the interface between the two monomeric subunits of DexAS^87A/D90A^ (interface 2^D^) is different from interface 1 and interface 2, with five salt bridges between interface 2^D^ compared to six in interface 1 and four in interface 2 (Supplementary Fig. S6C), and a larger dimer interface of 1629 Å^2^ than interfaces 1 (1576 Å^2^) and 2 (1562 Å^2^). The existence of three distinct dimeric conformation suggests a degree of conformational flexibility in DexA, which would be affected by temperature, and facilitate the quaternary structure change to tetramer via hydrogen bonds at contact 2. Finally, we were able to fit the DexAS^87A/D90A^ structure into the cryo-EM maps of the dimers at 37°C and 25°C, and the tetramer via contract 2 in the map for the tetramer at 25°C (Supplementary Fig. S6D). In summary, our data suggest that contact 2 is disrupted at high temperatures, which causes DexA to move the tetramer–dimer equilibrium to exclusively a dimer.

### Probing the mechanism of the temperature-driven substrate switch

**Figure 5.**
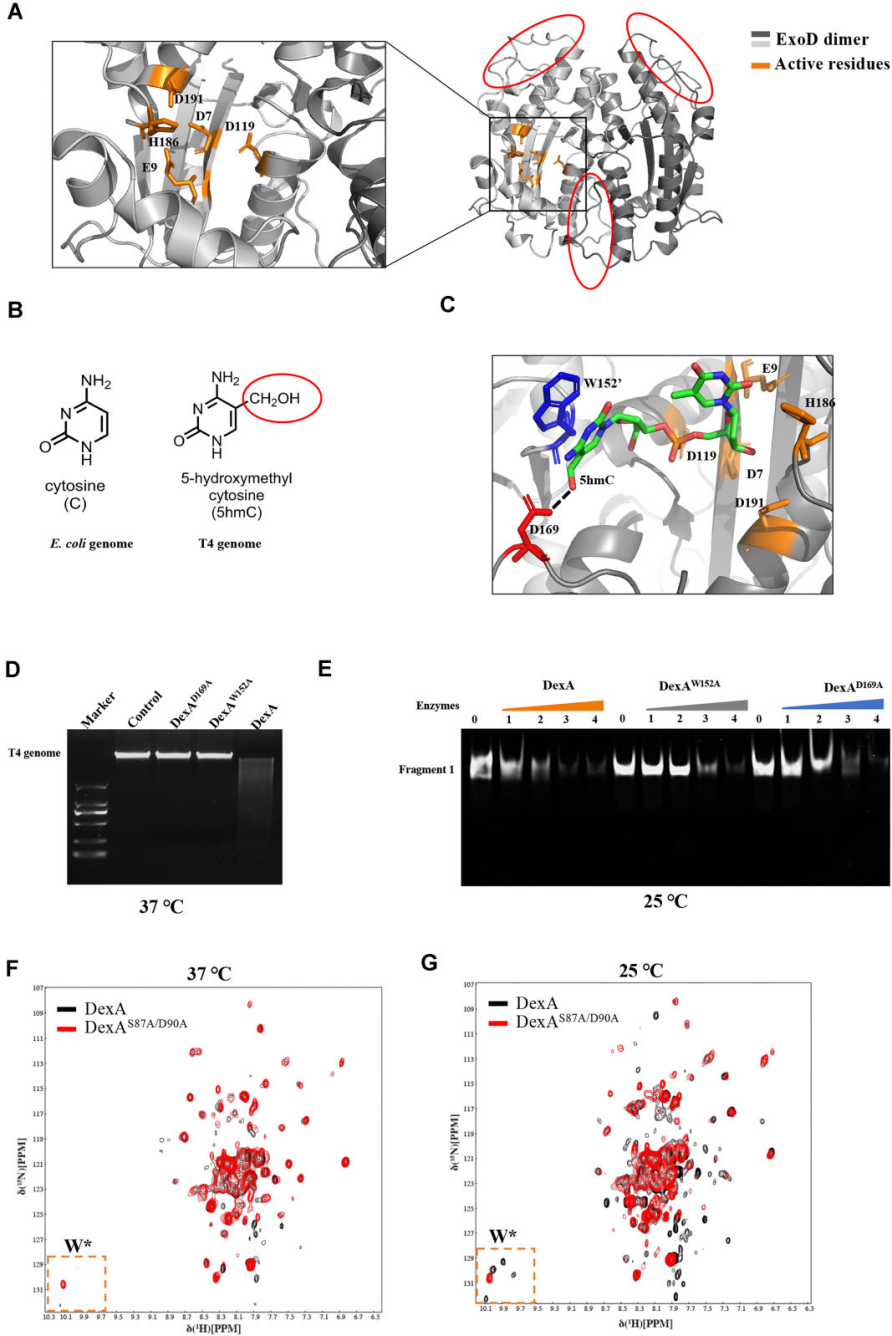
Conformational exchange allows DexA to recognize different DNAs. (**A**) The catalytic motif of the DexA dimer with all key residues labeled in orange: D7, E12, D119, D191, and H186. The red circles indicate extra loops in the DexA structure. (**B**) The structure of 5-hydroxymethyl modification of T4 cytosine compared to the unmodified counterpart in *E. coli*. (**C**) The catalytic motif of the DexA dimer contains 5hmC-modified DNA after simulation with all residues from panel (A), D169 and W152 from the other subunit labeled as the color scheme shows. The prime sign indicates that the residue is from the other subunit. (**D**) The agarose gel comparing the exonuclease activities of DexA, DexA^W152A^, and DexA^D169A^ using T4 genome as the substrate. The concentration of T4 genome 200 ng/μl (∼3 × 10^−3^ μM), while the concentrations for DexA and mutants were 1 μM. Control is T4 genome without any enzyme. (**E**) The TBE gel comparing the exonuclease activities of DexA, DexA^W152A^, and DexA^D169A^ using fragment 1 as the substrate at 25°C. The concentration of fragment 1 was 10 μM, while the concentrations for DexA and the mutants were 5, 10, 15, and 20 μM, corresponding to labels 1–4, respectively. The reaction temperature is labeled below the gels. (**F**) Overlay of 2D ^1^H–^15^N HSQC spectra of DexA and DexA^S87A/D90A^ recorded at 37°C. (**G**) Overlay of 2D ^1^H–^15^N HSQC spectra of DexA and DexA^S87A/D90A^ recorded at 25°C. The side chains of the putative tryptophan residues are indicated in red square.

To explore the temperature dependence of DexA nuclease activity, we first compared the DexA dimeric structure with other exonucleases. Using structural homology comparison server Dali [32], we found that human mitochondrial RNA exoribonuclease 2 (Rexo2) [33] is the closest structural homologue to the dimer. Rexo2 is also a homodimer whose exoribonuclease sites are formed jointly by the residues M50, L53, W96, and H100 from one subunit with H163 and Y164 from the other (Supplementary Fig. S7A and B). These residues are not well conserved in DexA and there would be insufficient space in the substrate binding site to accommodate the additional hydroxymethyl group of modified T4 DNA; therefore, it is unlikely that these residues are responsible for the unique exonuclease activity of DexA. The ϵ subunit of *E. coli* DNA Pol III, a well-known 3′–5′ exonuclease, is the second closest homologue of DexA [34]. Although ϵ subunit is a monomer, all the key residues in the active site, i.e. D12, E14, D103, D167, and H162, are conserved in DexA (Fig. 5A and Supplementary Fig. S7C). In DexA, there are two additional loops compared to the ϵ subunit, including one located at the monomer–monomer interface (loop 1 as shown in Supplementary Fig. S7C). Furthermore, there is no evidence showing that C5 of cytosine participates in the recognition process, as revealed by other *E. coli* exonucleases [27]. Thus, it is more likely that this set of residues are responsible for the exonuclease activity of DexA with additional interactions between 5hmC and adjacent residues in the active site.

To verify our speculation, we performed MD to find potential residues that may contribute to recognizing the 5hmC modification (Fig. 5B). We first constructed the DexA/DNA complex by replacing ϵ subunit with DexA based on the complex structure of ϵ/DNA (PDB ID: 1J54) and performed a simulation. We then modified the substrate from cytosine to 5hmC for another round of simulation at 37°C. As demonstrated in Supplementary Movie M3, within 5 ns unmodified DNA moves away from the initial position required for exonuclease activity. In contrast, 5hmC-modified DNA forms additional interactions with D169 of the same subunit and W152 of the adjacent subunit and remained within the active site throughout the simulation (Fig. 5C and Supplementary Movie M4). The additional hydrogen bond network and ring–ring pi stacking allows the DexA provide specificity for 5hmC binding. To confirm the role of W152 and D169, we constructed two mutants—DexA^W152A^ and DexA^D169A^—and tested their exonuclease activity. The results showed both mutants lose nuclease activity against T4 genome but retain the activity for unmodified DNA, supporting the MD results (Fig. 5D and E). However, glc-HMC-modified DNA also moves away from the initial position required for exonuclease activity during the simulation (Supplementary Movie M5), suggesting that DexA does not recognize the further glucosylation. Although this observation was not expected, there appears to be sufficient space in the catalytic site to accommodate the glc-HMC modification and any hydrogen bonds involving the HMC moiety can be satisfied by hydroxyls from glucose. While it is conceivable that the MD simulations are not capturing the full range of conformations for glc-HMC, they were instrumental in identifying key residues for our experimental mutational analyses.

Unmodified DNA becomes the preferred substrate as DexA populates the tetramer state at 25°C. It is therefore conceivable that the dimeric structure is altered in the context of the tetramer, which would restrict entry for the larger 5hmC but permits entry of smaller unmodified DNA. To explore this, we examined the temperature effects on the conformational properties of DexA by monitoring NMR spectra. At 37°C, the ^1^H–^15^N HSQC spectrum of the wild type is very similar to that of dimeric mutant DexA^S87A/D90A^, supporting the observation that the dimeric state is the predominate form at higher temperature (Fig. 5E). Another notable feature is that the lack of amide cross-peaks for many residues, due to peak broadening effects in both wild-type and mutant spectra, which is likely due to the conformational exchange on an intermediate timescale. Strikingly, more peaks appear in the wild-type tetrameric state at lower temperature of 25°C despite the presence of two species from the dimer–tetramer equilibrium (Fig. 5F). Four putative peaks corresponding to side chain H–N cross-peaks of all four tryptophan residues are visible in the lower temperature spectrum, while only one is visible at the same contour level in the mutant or higher temperature wild-type spectrum. This suggests that the conformation flexibility within the dimeric state is dampened in the tetramer at the lower temperature, 25°C. It is conceivable that at low temperatures the dimer is stabilized in a single conformation by tetramer formation. In summary, we propose that the temperature-driven quaternary structure change enables DexA to discriminate between modified T4 DNA and *E. coli* DNA.

### Bacteria produce DexA homologues that only recognize 5hmC-DNA

**Figure 6.**
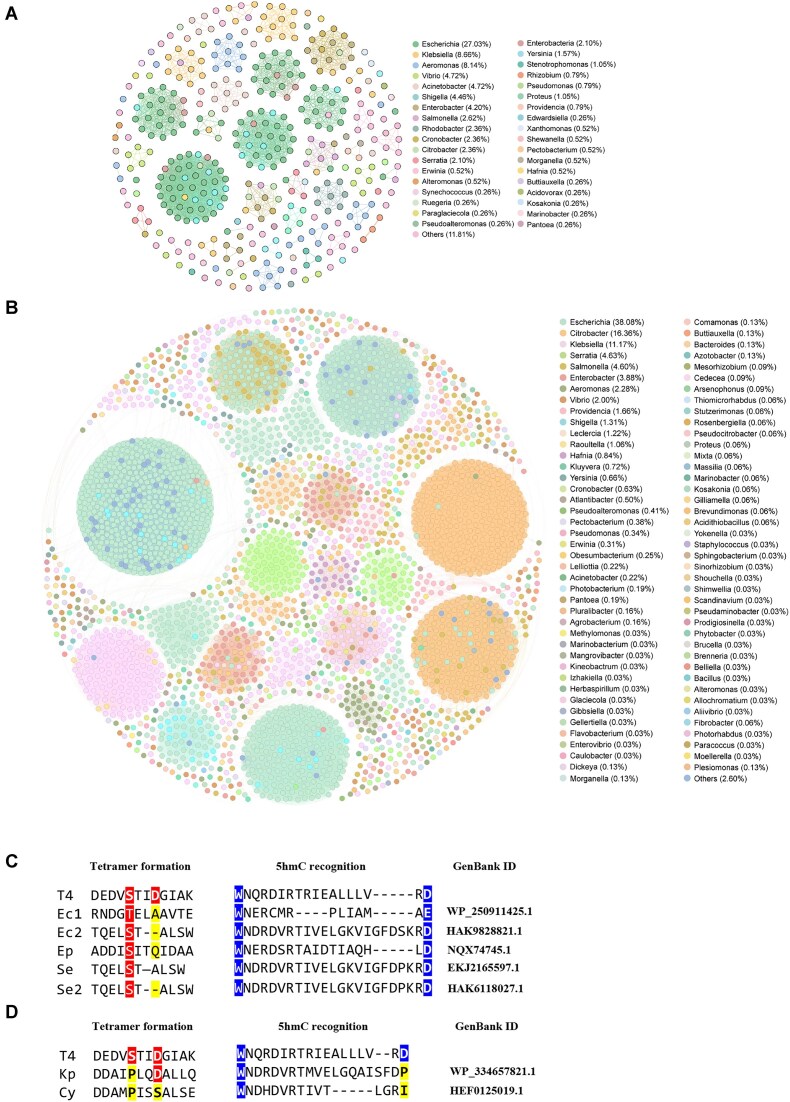
The conservation of DexA among bacteria and phages. (**A**) A visualized network of associations and distributions showing the hosts of 381 phages with unambiguous phage exonuclease sequences. The hosts of the phages, which belong to the Caudoviricetes class but lack specific taxonomy and host information, are noted as “other.” (**B**) A visualized network of associations and distributions showing 3196 bacteria with nonredundant DexA homologues. The nodes were colored based on different taxonomic groups at the host genus (A) and genus (B) levels, with the abundance of each genus indicated. (**C**) The sequence alignment of T4 DexA with the DexA homologues in selected bacteria strains. The residues that are responsible for 3′–5 exonuclease activity are fully conserved. The residues for tetramer formation are highlighted in red, while the yellow indicates the absence of the residue that is required for forming the salt bridge or 5hmC recognition. The residues that are required for 5hmC recognition are highlighted in blue. Ec: *E. coli*; Ep: *Epibacterium* sp.; and Se: *Salmonella enterica*. (**D**) The sequence alignment of T4 DexA with the DexA homologues in selected bacteria strains. The residues that are responsible for 3′–5 exonuclease activity are fully conserved. The residues for tetramer formation are highlighted in red, while the yellow indicates the absence of the residue that is required for forming the salt bridge. The residues that are required for 5hmC recognition are highlighted in blue. Kp: *Klebsiella pneumoniae*; and Cy: *Citrobacter youngae*.

To determine whether the HREX strategy is commonly used by phages, we performed a BLAST search using the DexA sequence. Our results suggest that DexA is highly conserved among phages infecting a broad spectrum of bacterial hosts (Fig. 6A), with *Escherichia* (26.77%) and *Klebsiella* (8.66%) representing the most common hosts. Notably, many of these phages have been reported to carry modified DNA, as revealed by recent high-throughput sequencing analyses [35]. Strikingly, it is also highly conserved among bacteria (Fig. 6B). Bacterial DexA homologues can be found in a broad range of bacteria, including γ-proteobacteria, α-proteobacteria, β-proteobacteria, Bacteroidetes, Actinobacteria, Acidithiobacillia, Bacilli, and Fibrobacteres, spanning 83 genera, with the most prevalent being *Escherichia (*38.08%), *Citrobacter* (16.36%), and *Klebsiella* (11.17%) within γ-proteobacteria. In fact, its homologues can be found in all major Gram-negative bacterial pathogens, including *Acinetobacter baumannii*, *Klebsiella pneumoniae*, *Pseudomonas aeruginosa*, and *E. coli*. Notably, the hosts of DexA-containing phages overlap very well with the bacteria containing DexA homologues, suggesting the possibility of bacteria producing an exonuclease that digests invading phages with 5hmC-modified DNA.

Since DexA^S87A/D90A^ is a mutant that resembles the dimeric state that cleaves 5hmC-modified DNA, bacteria producing DexA^S87A/D90A^ homologues, in principle, would be granted immunity to these phages. To verify this speculation, we scanned the sequences of DexA homologues in bacteria, focusing on the conservation of the residues responsible for 5hmC recognition and the absence of the residues for tetramer formation. The results show that most bacterial DexA homologues contain the residues that are responsible for tetramer formation. To illustrate the feature of the bacterial homologues, we selected five representative DexA-like sequences from different bacterial strains and compared the residues that are responsible for the tetramer formation and 5hmC recognition (Fig. 6C). In the five bacterial DexA-homologue sequences, the tryptophan and the negatively charged residue, aspartic acid or glutamic acid, predicted to be required for 5hmC recognition are conserved. However, the mutual hydrogen bonds for tetramer formation would be disrupted due to absence of D90 at equivalent positions. As all the residues required for exonucleolytic activity are fully conserved, the selected DexA homologue should be, theoretically, able to cleave 5hmC-modified DNA. Interestingly, the negative-charged aspartic acid is less conserved compared to the tryptophan in the 5hmC recognizing region (Fig. 6D). As D169 is likely to be key for 5-hydroxymethyl recognition, we propose that a substitution of or around D169 may allow HREX to recognize other types of phage DNA modifications at cytosine C5 or perhaps on other nucleotides [8].

### The bacterial HREX antiphage system

**Figure 7.**
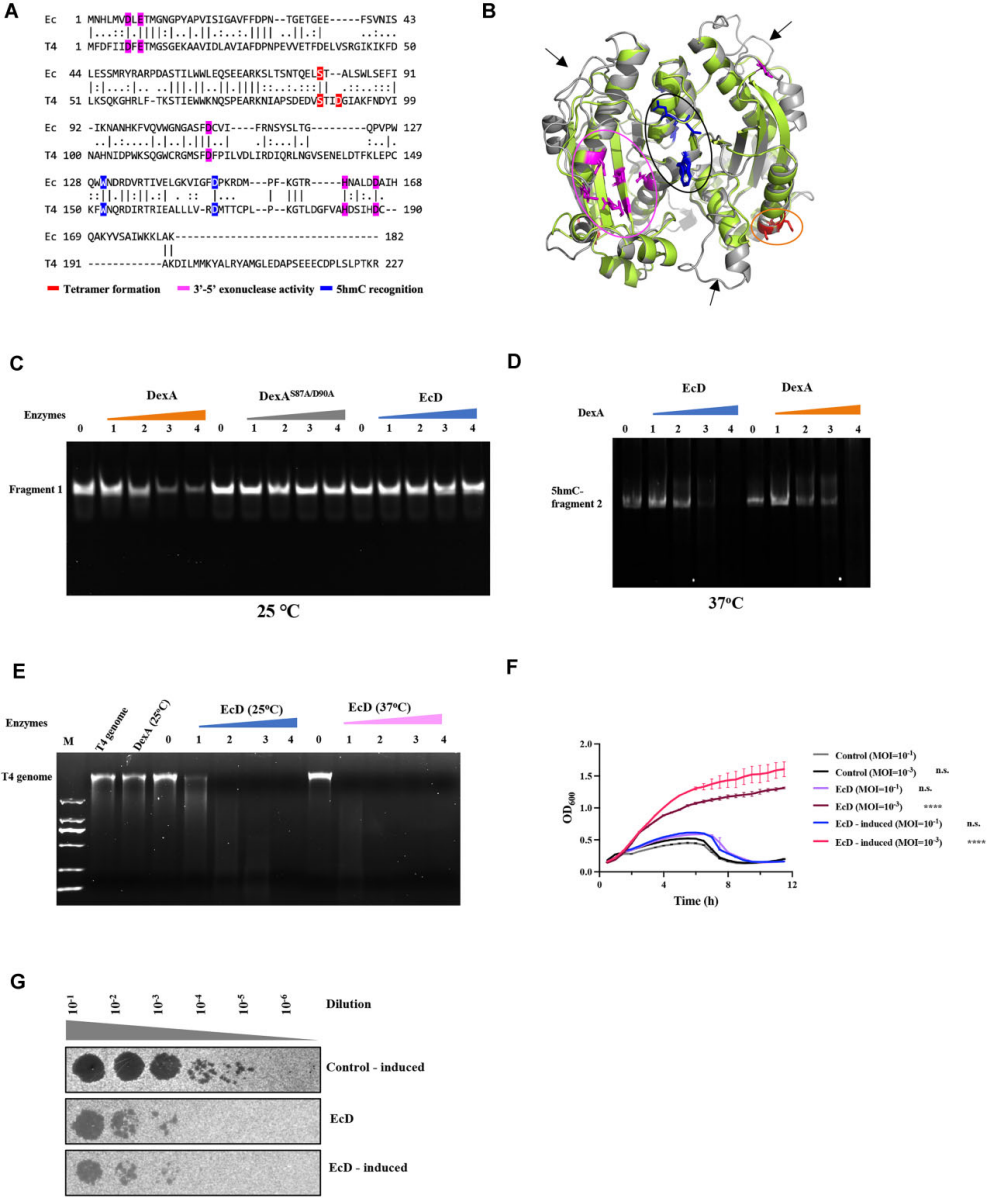
Bacteria HREX-based antiphage defense. (**A**) The sequence alignment DexA with EcD of *E. coli* (GenBank ID: HAK9828821.1). The residues that are responsible for 3′–5 exonuclease activity are fully conserved (highlighted in gray). The residues for tetramer formation are highlighted in red and those that are required for 5hmC recognition are highlighted in blue. (**B**) The structure overlay of DexA (AB subunit as shown in Fig. 3A and colored in gray) and the EcD model (lime). The residues for tetramer formation are highlighted in red and those that are required for 5hmC recognition are colored in blue. The arrows indicate the extra loop and helices regions only present in DexA. (**C**) The TBE gel comparing the exonuclease activities of DexA, DexA^S87A/D90A^, and EcD using fragment 1 as the substrate at 25°C. The concentration of fragment 1 was 10 μM, while the concentrations for DexA and the mutants were 5, 10, 15, and 20 μM, corresponding to labels 1–4, respectively. (**D**) The TBE gel comparing the exonuclease activities of EcD and DexA using 5hmC-modified fragment 2 as the substrate at 37°C. The concentration for 5hmC–fragment 2 was 0.5 μM, while the concentrations for EcD and DexA were 0.25, 1, 2, and 4 μM, corresponding to labels 1–4, respectively. (**E**) The agarose gel comparing the exonuclease activity assay of DexA and EcD at 25°C and 37°C using T4 genome as the substrate. The concentration of T4 genome was 200 ng/μl (∼3 × 10^−3^ μM), while the concentration of DexA was 1 μM, and the concentrations for EcD were 0.25, 0.5, 1, and 2 μM, corresponding to labels 1–4, respectively. (**F**) The growth curves of T4 infecting *E. coli* with plasmid-borne EcD at 37°C. The control is *E. coli* cells bearing the empty circular plasmid. Error bars show SEM; n.s., not significant (*P* > .05), ^****^P < .0001; two-way ANOVA, *n* = 3. (**G**) The phage plaque assay showing the T4 plaque formation under the effect of EcD.

To demonstrate the potential antiphage effect of the bacterial DexA dimer state homologue, we selected one (termed EcD) from a representative *E. coli* strain (Fig. 7A). In the EcD sequence, residues for the exonuclease activity are fully conserved, as are those for the 5hmC recognition. However, the aspartic acid (D90 as in DexA) that is required for the formation of the tetramer is replaced by alanine in EcD (A78 as in EcD). This suggests that EcD is a DexA^S87A/D90A^ homologue that may offer *E. coli* the protection against T4. We then built a homologous model of EcD using SWISS-MODEL [36] based on DexA^S87A/D90A^ structure, and compared it with its DexA subunit AB (Fig. 7B). In the EcD model, residues responsible for exonuclease activity and 5hmC recognition occupy similar positions within the catalytic site; however, several surface loops and helices present in DexA are absent in the *E. coli* counterpart. Notably, in the nucleolytic activity assay, EcD loses its nucleolytic activity against unmodified dsDNA at 25°C, confirming that it would not be cytoxic (Fig. 7C). Meanwhile EcD exhibited a high level of nucleolytic activity against the T4 genome at 25°C, unlike DexA that does not at this temperature (Fig. 7E). Given the conservation of catalytic residues, these results suggest that the inability of EcD to form a tetramer underlies its selective activity—inactive on unmodified host DNA while active against 5hmC-modified T4 DNA. In the infection growth assay, the growth curves of T4-infecting *E. coli* suggest the existence of a defense system, with plasmid-borne EcD granting the *E. coli* immunity against T4 infection (Fig. 7E). The fact that EcD can protect bacterial cells, even without IPTG due to leaky expression, suggests that it is highly effective at digesting invading T4 genes. Similarly, the plaque assay also suggests the existence of the EcD antiphage system in *E. coli* (Fig. 7F). In summary, EcD, a homologue of *E. coli* DexA, retains the ability to specifically digest the T4 genome through the HREX strategy, highlighting its role in recognizing and cleaving 5hmC-modified DNA. This strategy may not be limited to T4 but could represent a broader defense mechanism employed by bacteria to distinguish and degrade modified phage DNA, offering a targeted yet adaptable approach to combating phages.

## Discussion

Combining major structural analysis tools, we have described a mechanism that allows T4 phage to recognize two distinct substrates under different environmental conditions. Cryo-EM and X-ray crystallography revealed a core dimeric form of DexA that undergoes a transformation to tetrameric form at lower temperatures, through a new interface stabilized by S87 and D90. As the temperature drops, stabilized tetrameric DexA and a dampening of the dynamics likely play a role in restricting its specificity to unmodified DNA. Preventing the transition by mutation, as seen in DexA^S87A/D90A^, keeps DexA into a state where it specifically utilizes 5hmC-modified DNA as its substrate. At higher temperatures, DexA multimerization is reduced, which is detrimental to its ability to cleave unmodified DNA. Furthermore, although bacterial exonucleases capable of cleaving both ssDNA and dsDNA are not well described, such examples can be found among endonucleases. *Escherichia coli* endonuclease DNase I can use both dsDNA and ssDNA as substrates, adapting to different DNA conformations to allow it to bind and cleave both forms nonspecifically through hydrolysis [37]. While DNase I prefers dsDNA, it can still cleave ssDNA, though different conditions may be required for optimal activity. DexA also has such characteristics, e.g. nonspecific DNA binding and conformational adaptation, similar to DNase I, but it prefers ssDNA over dsDNA. This feature allows DexA to effectively digest host DNA at break sites, such as sticky ends or double-strand breaks, caused by various internal nucleases and external stress factors [38].

The exchange of DexA between a dimer and tetramer with distinct functions is reminiscent of the the metamorphic protein KaiB. The cyanobacterial protein KaiB switches between monomer, dimer, and tetramer states [39]. Metamorphic proteins defy the one-sequence, one-fold paradigm in structural biology and only about five have been well characterized [40, 41]. These usually adopt two or more folded structures or states to offer functional diversity. Although DexA does not fit the category of true metamorphic protein as it undergoes a quaternary structural transition and not a fold change, its temperature-dependent switch is closely linked to phage biology. The optimal temperature for T4 is 37–42°C, similar to that of its host *E. coli* [42]. At 37°C, the group I intron homing of T4 phage is a nonessential yet beneficial biological activity that drives the transfer of introns as a source of genetic novelty [43]. Thus, the dimer state induced by optimal temperature is designed for intron homing to better phage adaptation. This, however, would become a burden during nonideal growth conditions. Indeed, it has been demonstrated that T4 reduces the self-splicing efficiency of group I introns under unfavorable conditions, which are frequently encountered by natural phage populations [25]. The quaternary structure switch exhibited by DexA is in line with the phenotype observed at low temperatures, during which substrate preference shifts from 5hmC-modified T4 DNA to *E. coli* DNA to scavenge resource. The low-temperature-induced tetramer enables phage to turn to recycling host DNA for its own nucleotide needs instead of the nonessential homing event. Thus, DexA mechanism represents a smart approach for phage to maximize the utilization of a single gene product and provides a competitive advantage over other *E. coli* phages.

To evade the bacterial defense, many bacteriophages modify their nucleotides to gain resistance to the cleavage by various restriction enzymes that bacteria produce [44]. The classic RM system, which normally comprises two contrasting enzymatic activities: an endonuclease and a methyltransferase, is one of the most common bacterial responses that against the modification strategy [45]. In type IV RM systems where the methyltransferase is absent, the REase can also recognize a particular sequence and cleave modified phage DNA [46]. EcD proteins specialize in the recognition and cleavage of 5hmC-modified phage DNA, representing a new example of a bacterial strategy to distinguish and eliminate phage genomes. Although both EcD (or the HREX system) and the RM system function by cleaving phage DNA, they do so through distinct mechanisms. EcD acts as a modification-dependent exonuclease, providing 5hmC-specific defense without recognizing specific sequence. In contrast, the RM system offers sequence-specific protection through the coordinated action of restriction endonucleases and methyltransferases. Together, the two distinct systems based on cleaving phage DNA highlight the diverse and evolving strategies bacteria employ to survive phage attacks in the ongoing evolutionary arms race. As D169 is suggested to be involved in 5-hydroxymethyl recognition, we also propose that a substitution of or around D169 may allow DexA-like protein to recognize other types of phage DNA modifications at cytosine C5 or perhaps even on other nucleotides [8]. Furthermore, based on our phylogenetic analysis, the DexA-like proteins are widely present in γ-bacteria, yet overlooked.

**Figure 8.**
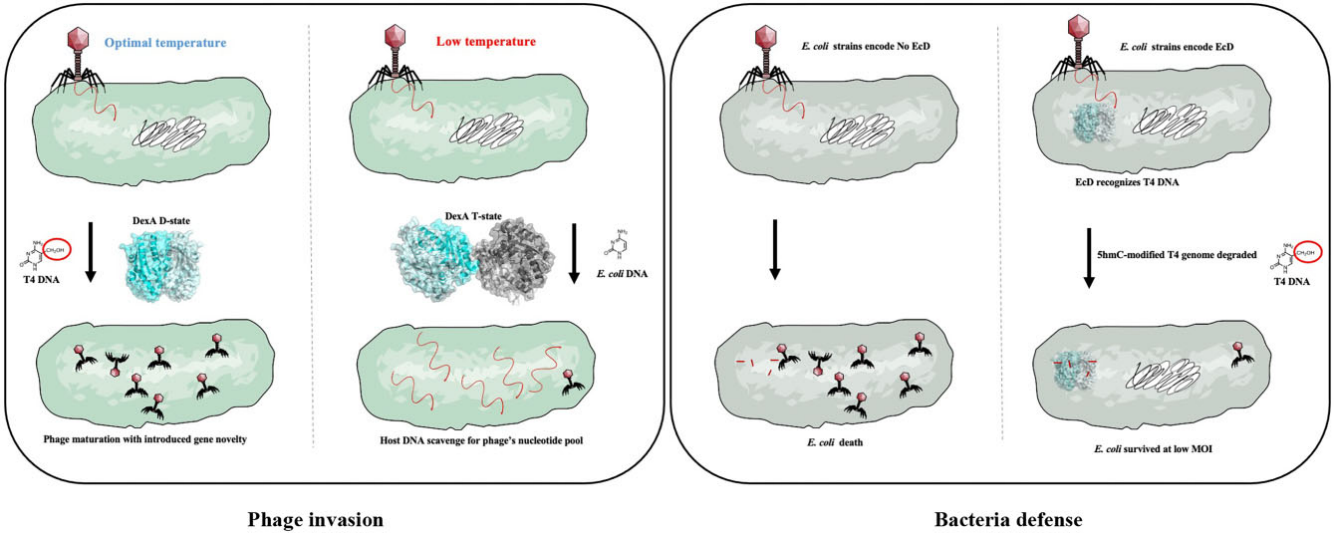
HREX—a DNA discrimination strategy shared by bacteria and phages. Illustration of the biological role of DexA that undergoes temperature-sensing fold switch in T4, and *E. coli* EcD for its self-defense against T4-like phage.

As bacteria adapt the phage temperature-driven DNA discrimination strategy system by tuning it solely for recognizing modified DNA, the HREX strategy is therefore a rare example of one employed by both phage and bacteria during co-evolution (Fig. 8). Unfortunately, DexA homologues are not present in the *E. coli* K12 strains that are commonly used as tools in molecular biology and cell factories for protein production. T4 or T4-like phage contamination is a serious issue, in large-scale industrial fermenters and laboratory-scale setting using *E. coli* [47]. We therefore propose that the *ecD* gene should be incorporated into the *E. coli* K12 strains or be cloned into a vector to reduce T4-like phage contaminations in laboratories and industries [48].

## Supplementary Material

gkaf501_Supplemental_Files

## Data Availability

Structural coordinates of DexA and DexA^S87A/D90A^ have been deposited in Protein Data Bank under accession codes 8KEL and 8KEN, respectively.
