# Supplementary material for: A temperature-driven DNA discrimination strategy to distinguish E. coli DNA and phage 5hmC-modified DNA
Source: Nucleic Acids Res. 2025 Jun 11;53(11):gkaf501. doi: 10.1093/nar/gkaf501 (PMC12153337; doi:10.1093/nar/gkaf501)
Supplement: gkaf501_Supplemental_Files [file gkaf501_supplemental_files.zip › s_fig_table12.pdf]

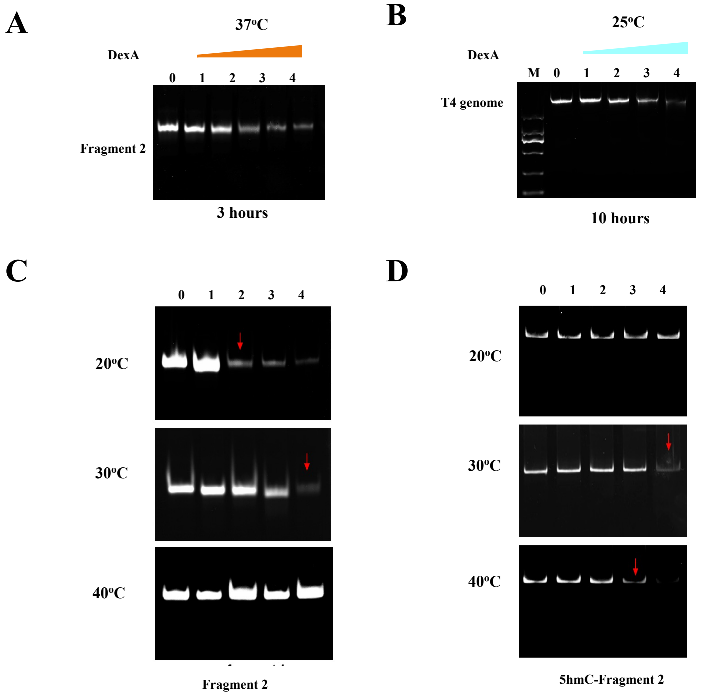

Supplementary figure 1: exonuclease activity assay under extended time

(**A**) The TBE gel showing the 3-hour exonuclease activity assay of DexA using fragment 2 as the substrate. The concentration of unmodified fragment 2 was 10 µM, while the concentrations for DexA were 5, 10, 15, and 20 µM, corresponding to labels 1 to 4, respectively. (**B**) The TBE gel showing the 8-hour exonuclease activity assay of DexA using T4 genome as the substrate. The concentration of unmodified fragment 2 was 10 µM, while the concentrations for DexA were 5, 10, 15, and 20 µM, corresponding to labels 1 to 4, respectively. (**C-D**) Top to bottom: The TBE gels showing the exonuclease activity assays of DexA using fragment 2 (100 bp) and 5hmC-fragment 2 as the substrates at 20°C, 30°C and 40°C, respectively. The concentration of unmodified fragment 2 was 10 µM, while the concentrations for DexA were 5, 10, 15, and 20 µM, corresponding to labels 1 to 4, respectively. And the concentration for 5hmC-fragment 2 was 0.5 µM, while the concentrations for DexA were 0.5, 1, 2, and 4 µM, corresponding to labels 1 to 4, respectively.

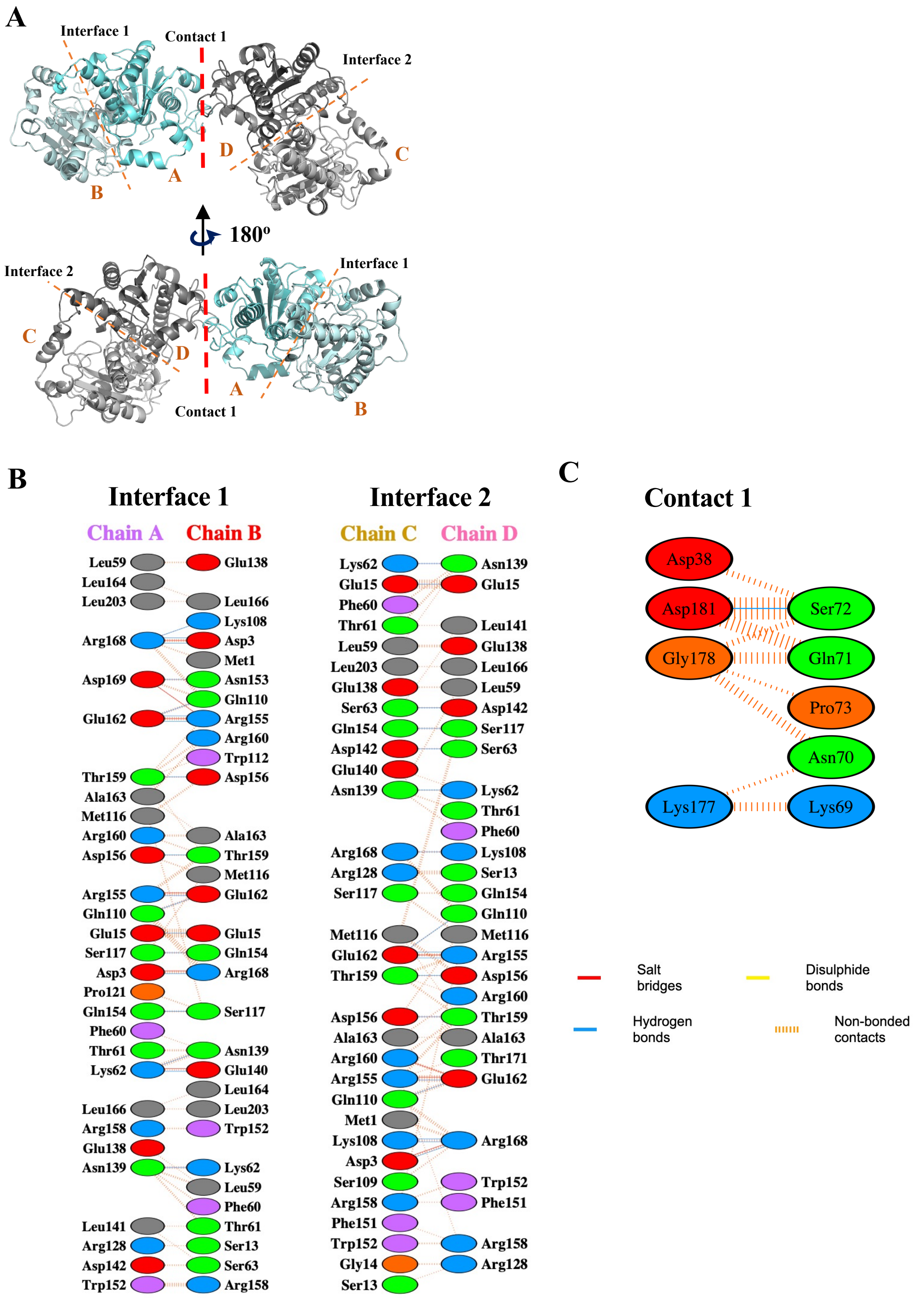

Supplementary figure 2: Crystal structure of DexA

(**A**) The cartoon representation of the crystal structure of DexA displayed in 180º rotation, with subunits A-D labelled in orange. (**B**) The types of interactions and the residues involved at interface 1 and 2. (**C**) The types of interactions and the residues involved at contact 1. The different interaction types are shown in different line as illustrated at the bottom. Residue are also coloured according to their biochemical property: Blue = positive (H,K,R); Red = negative (D,E); Green = neutral (S,T,N,Q); Grey = aliphatic (A,V,L,I,M); Purple = aromatic (F,Y,W); Orange = Pro&Gly. The images are generated by PDBsum.

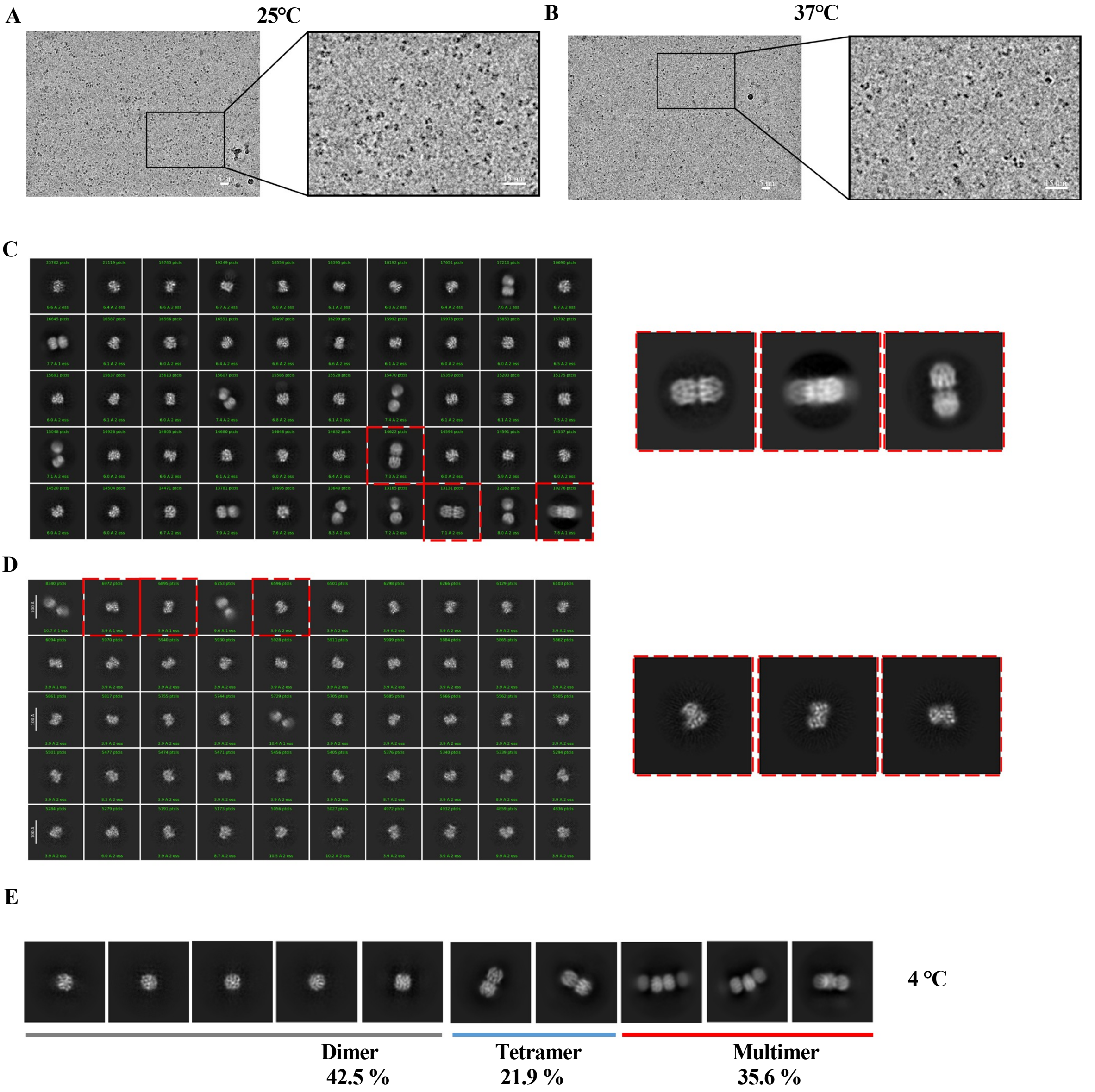

Supplementary figure 3. Cryo-EM analysis of the DexA incubated at different temperature. (A-B) Representative micrograph of DexA at 25°C (**A**) and 37°C (**B**) with insets on the right. **(C-D)** 50 reference-free 2D classes of DexA at 25 °C (**C**) and 37 °C (**D**). Representative classes of DexA tetramer at 25°C and 37°C are labeled with red box and displayed below for comparison. **(E)** 2D classification of DexA at 4 ^{o}C shown it exists in a mixtures of dimer, tetramer and multimers.

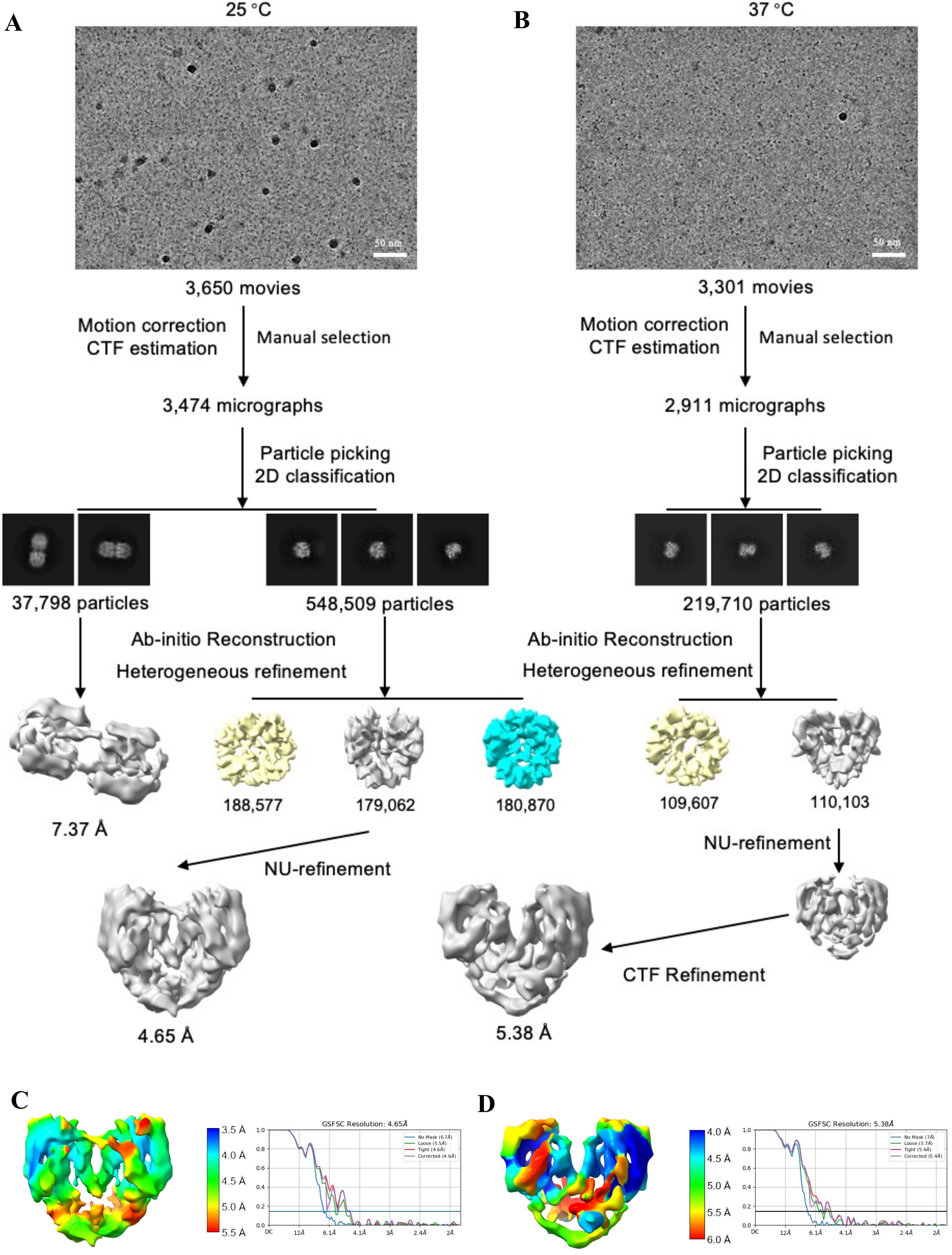

Supplementary figure 4. Cryo-EM analysis of DexA datasets.
(**A-B**) Flow chart of cryo-EM data analysis of DexA at 25 °C and 37°C.
(**C-D**) Local resolution of the map and Golden Standard Fourier Shell Correlation (GSFSC) curve of DexA at 25 °C and 37°C.

A

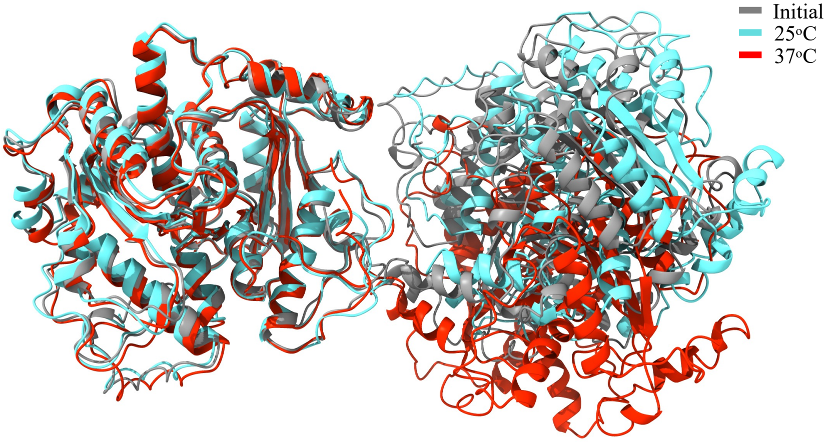

B

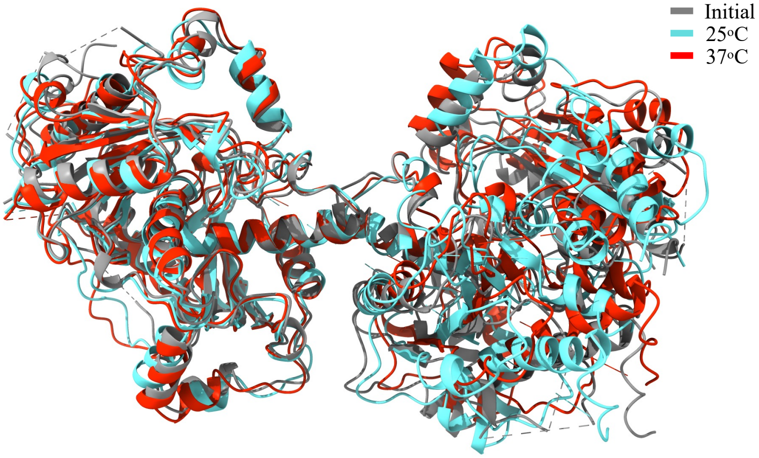

Supplementary figure 5: Molecular dynamic simulation of the tetrameric crystal structure **(A)** The overlay of the initial structure, the structure simulated at 25 °C and the structure simulated at 37 °C, using tetramer formed at contact 1. **(B)** The overlay of the initial structure, the structure simulated at 25 °C and the structure simulated at 37 °C, using tetramer formed at contact 2.

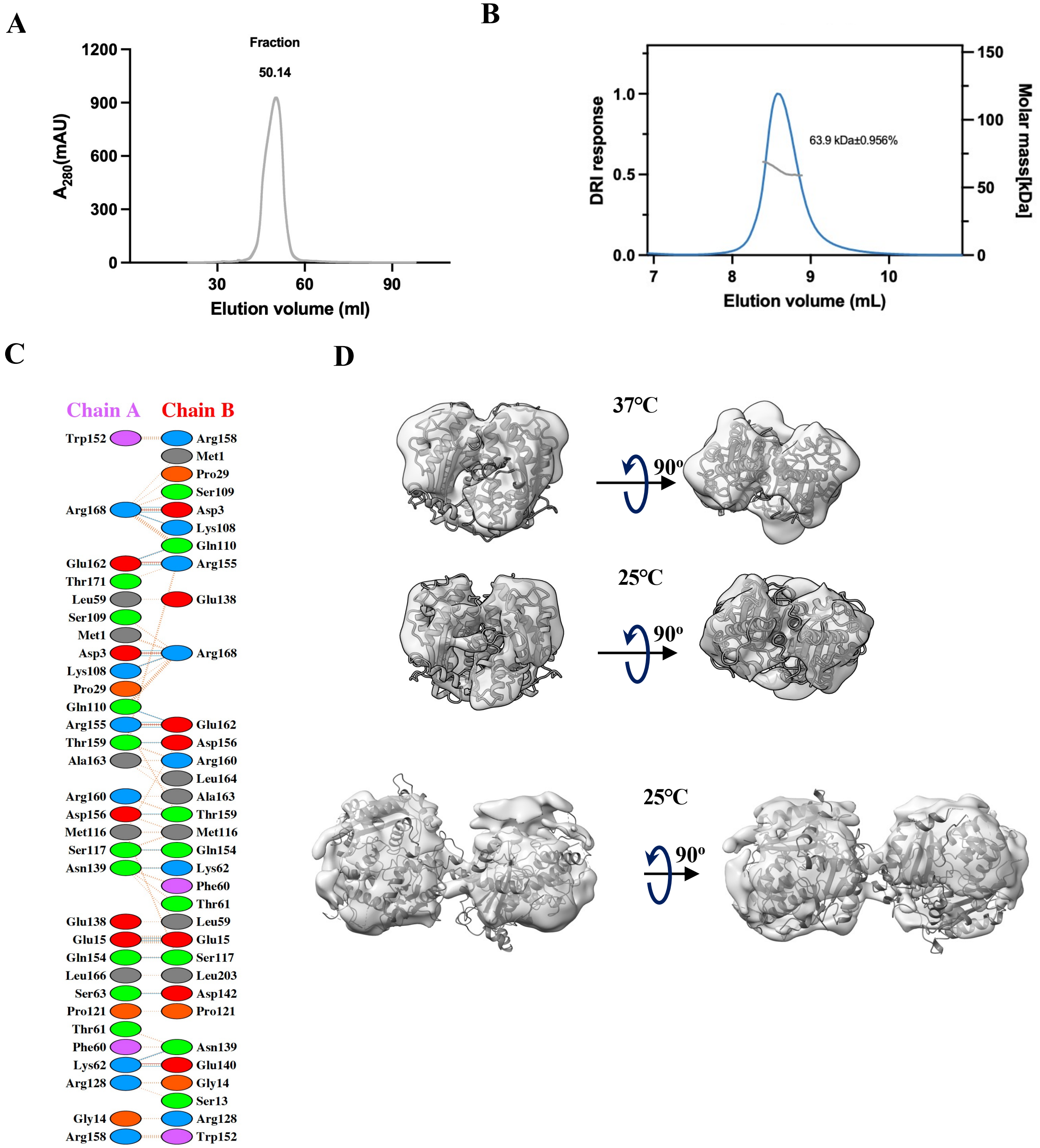

Supplementary figure 6: Characterization of the DexA$^{S87A/D90A}$ mutant

(A) The ion exchange chromatographic profile of DexA$^{S87A/D90A}$ using a Mono Q column. (**B**) The SEC-MALS profiles of DexA$^{D38A/H186A}$ and calculated MW (20 µM before injection) at 25°C. (**C**) The interactions and residues involved at interface 2^{D}. The different interaction types are shown in different line as illustrated at the bottom. Residue are also coloured according to their biochemical property: Blue = positive (H,K,R); Red = negative (D,E); Green = neutral (S,T,N,Q); Grey = aliphatic (A,V,L,I,M); Purple = aromatic (F,Y,W); Orange = Pro&Gly. The image is generated by PDBsum. **(D)** The dimeric DexA$^{S87A/D90A}$ structure fitted into the cryo-EM maps of DexA dimer prepared at 37 ^{o}C ; the dimer and the tetramer at 25 ^{o}C .

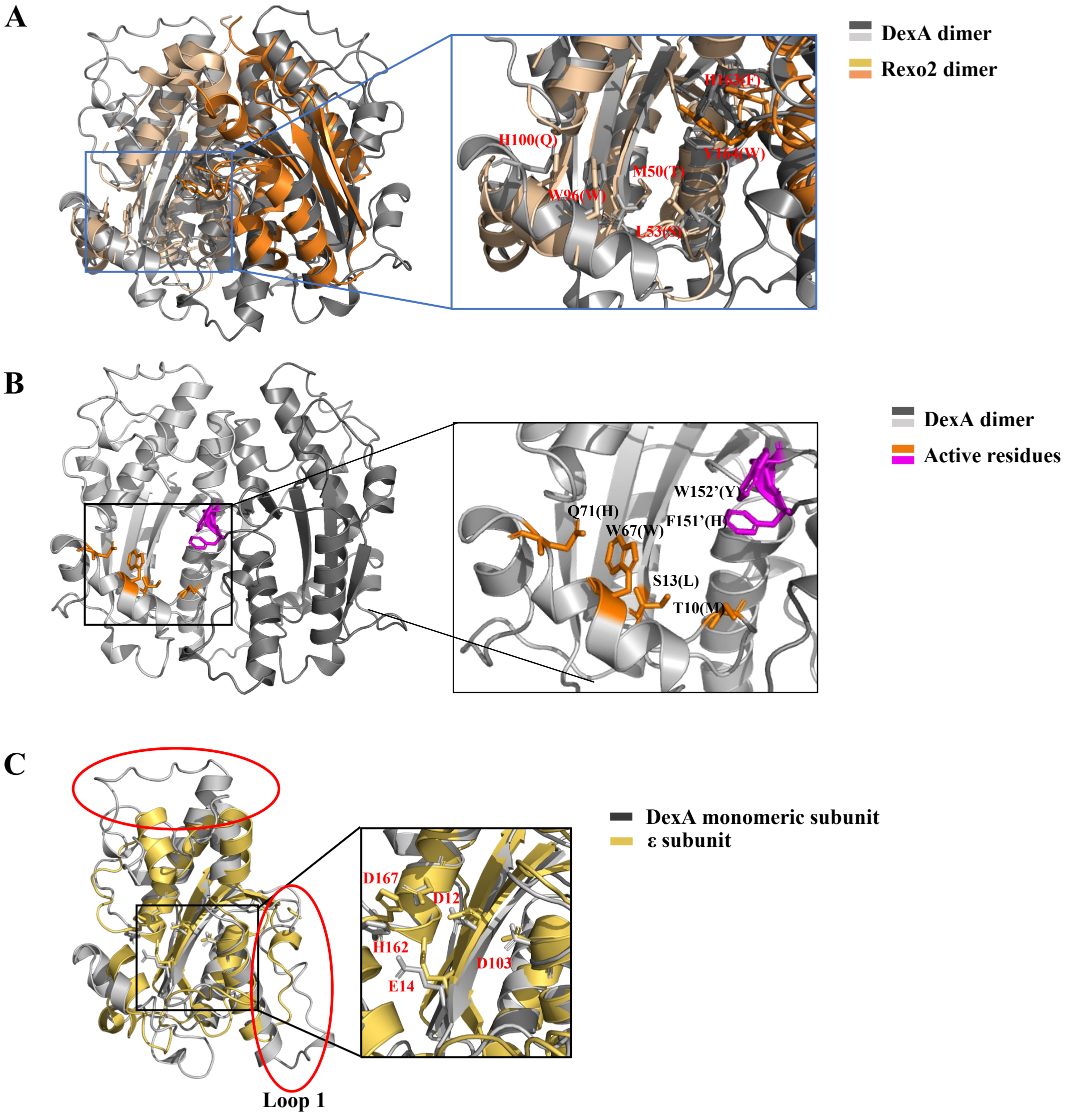

Supplementary figure 7: Concentration effects on the protein size

(A) The structure overlay of DexA dimer and human RNA exoribonuclease 2 (PDB ID: 6J7Y). All active residues M50 (T10), L53 (S13), W96 (W67) and H100 (Q71) from one subunit, and H163 (F151) and Y164 (W152) from the other subunit for Rexo2 are labeled. The corresponding residue numbers of DexA are showing in bracket. The corresponding residue of DexA are showing in bracket. **(B)** The structure of DexA dimer with all putative catalytic residues labelled. The prime sign indicates that the residue is from the other subunit. All active residues The corresponding residue of Rexo2 are showing in bracket as shown in **(A)**. **(C)** The structure overly of D-state and *E. coli* DNA Pol III ε subunit (PDB ID: 1J54). All key residues in the active site - D12 (D7), E14 (E12), D103(D119), D167 (D191) and H162 (H186) are labeled. The corresponding residue numbers of DexA are showing in brackets. The red circles indicate extra loops in the DexA structure.

Table S1. Oligonucleotides list

	Oligomer (5' - 3')
DexA_F_Pet 46	GATGACGACGACAAGATGTTTGATTTTATTATAGATTTTGAAAC
DexA_R_Pet 46	TGAGGAGAAGCCCGGTTATCGTTTTGTTGGAAGAGATAG
DexA_S87A /D90A_F	ATCGCCGGTATTGCAAAATTTAATGATTAC
DexA_S87A /D90A_R	AGTGGCTACATCTTCATCGGATGG
DexA_D169 A_F	GCTTGTTCGTGCGATGACAACGTG
DexA_D169 A_F	AGAAGTGCTTCAATTCTAG
DexA_D38A /H186A_F1	TGAAACATTCGCGGAATTAGTTTCACG
DexA_D38A /H186A_R1	ACTACTTCTGGATTAGGG
DexA_D38A /H186A_F2	ATTCGTTGCAGCGGATTCTATTCATGACTGTG
DexA_D38A /H186A_R2	CCATCTAAAGTTCCCTTTG
DexA_D181 A/S72A_F1	GGAACTTTAGgcgGATTCGTTGCAC
DexA_D181 A/S72A_R1	CTTTGGAAGAGGACAC
DexA_D181 A/S72A_F1	AAGAATCAATgcgCTGAAGCTCGAAAAAATATTGC
DexA_D181 A/S72A_F1	CCACCATTCGATAGTAC
DexA_F_pB AD	CATCACAGCAGCGGCATGTTTGATTTTATTATAGATTTTGAAACAATGGG
DexA_R_pB AD	AAAACAGCCAAGCTTATCGTTTTGTTGGAAGAGATAGAGG
EcD_F	GATGACGACGACAAGATGATTGACACCGAAAC
EcD_R	TGAGGAGAAGCCCGGTTATCATATGTTCAGGAATGGT
Oligo 1	CTCATATGAATCACGTAGAACAAT
Oligo 2	ATTGTTCTACGTGATTCATATGAG
Fragment 2_F	TAATATAGGAAGAGGTTATGATAGTAG
Fragment 2_R	TGGTGTGAGTATGGATGG
Fragment 2 (5'-3')	ATTATATCCTTCTCCAATACTATCATCAGTCTAGTCGTGGCAGTGATCGTCAT GGACTGGTGTTGTATGCCGGATAGCCGCAGGTAGGTATGAGTGTGGT; TAATATAGGAAGAGGTTATGATAGTAGTCAGATCAGCACCGTCACTAGCAG TACCTGACCACAACATACGGCCTATCGGCGTCCATCCATACTCACACCA

Table S2. Summary of data collection and refinement

	DexA	DexA$^{S87A/D90A}$
Space group	$P12_11$	$P2_12_12_1$
Unit cell (Å)	*a* = 84.548,<br>*b* = 76.285,<br>*c* = 108.009	*a* = 76.33,<br>*b* = 82.182,<br>*c* = 115.931
Wavelength (Å)	0.9789	0.9789
Resolution (Å)	28.3–2.881(2.984–2.881)	67.05–2.381(2.467–2.381)
Unique reflections[a]	30064 (3066)	29853 (2922)
Completeness[a] (%)	95.96 (99.16)	99.76 (99.86)
Intensity (I/σ) [a]	8.24 (1.89)	17.70 (2.12)
R_{merge}[a]	0.1389 (0.9416)	0.09151 (0.9996)
R_{work}[a]	0.2423 (0.3262)	0.2404 (0.3160)
R_{free}[a]	0.2662 (0.3492)	0.2847 (0.3705)
Average B factors (A^2)		
Protein	84.30	52.07
Ramachandran favored (%)	97.13	97.52
Ramachandran allowed (%)	2.63	2.48
Ramachandran outliers (%)	0.24	0
RMSD of bond length (Å)	0.002	0.007
RMSD of bond angle (°)	0.58	0.89

[a]Values in the parentheses refer to the highest resolution shell

Supplementary movie 1: MD simulations of DexA crystal structure at contact 1 at under 25 ^{o}C (Blue) and 37 ^{o}C (Red).

Supplementary movie 2: MD simulations of DexA crystal structure at contact 2 under 25 ^{o}C (Blue) and 37 ^{o}C (Red).

Supplementary movie 3: MD simulations of DexA/DNA complex at 37 ^{o}C.

Supplementary movie 4: MD simulations of DexA/5hmC-containing DNA fragment complex at 37 ^{o}C.

Supplementary movie 5: MD simulations of DexA/ glucosylated-5hmC-containing DNA fragment complex at 37 ^{o}C.
